# Advancing one health vaccination: In silico design and evaluation of a multi-epitope subunit vaccine against Nipah virus for cross-species immunization using immunoinformatics and molecular modeling

**DOI:** 10.1371/journal.pone.0310703

**Published:** 2024-09-26

**Authors:** Edward Coralde Banico, Ella Mae Joy Sinco Sira, Lauren Emily Fajardo, Albert Neil Gura Dulay, Nyzar Mabeth Obenio Odchimar, Alea Maurice Simbulan, Fredmoore Legaspi Orosco

**Affiliations:** 1 Department of Science and Technology, Virology and Vaccine Research Program, Industrial Development Technology Institute, Taguig City, Metro Manila, Philippines; 2 Department of Science and Technology, S&T Fellows Program, Taguig City, Metro Manila, Philippines; 3 Department of Biology, College of Arts and Sciences, University of the Philippines Manila, Manila City, Metro Manila, Philippines; University of Tabuk, SAUDI ARABIA

## Abstract

The resurgence of the Nipah virus (NiV) in 2023 has raised concerns for another potentially severe pandemic, given its history of high mortality from previous outbreaks. Unfortunately, no therapeutics and vaccines have been available for the virus. This study used immunoinformatics and molecular modeling to design and evaluate a multi-epitope subunit vaccine targeting NiV. The designed vaccine construct aims to stimulate immune responses in humans and two other intermediate animal hosts of the virus—swine and equine. Using several epitope prediction tools, ten peptides that induced B-lymphocyte responses, 17 peptides that induced cytotoxic T-lymphocyte (CTL) responses, and 12 peptides that induced helper T-lymphocyte (HTL) responses were mapped from nine NiV protein sequences. However, the CTL and HTL-inducing peptides were reduced to ten and eight, respectively, following molecular docking and dynamics. These screened peptides exhibited stability with 30 common major histocompatibility complex (MHC) receptors found in humans, swine, and equine. All peptides were linked using peptide linkers to form the multi-epitope construct and various adjuvants were tested to enhance its immunogenicity. The vaccine construct with resuscitation-promoting factor E (RpfE) adjuvant was selected as the final design based on its favorable physicochemical properties and superior immune response profile. Molecular docking was used to visualize the interaction of the vaccine to toll-like receptor 4 (TLR4), while molecular dynamics confirmed the structural stability of this interaction. Physicochemical property evaluation and computational simulations showed that the designed vaccine construct exhibited favorable properties and elicited higher antibody titers than the six multi-epitope NiV vaccine designs available in the literature. Further *in vivo* and *in vitro* experiments are necessary to validate the immunogenicity conferred by the designed vaccine construct and its epitope components. This study demonstrates the capability of computational methodologies in rational vaccine design and highlights the potential of cross-species vaccination strategies for mitigating potential NiV threats.

## Introduction

In September 2023, the resurgence of Nipah virus (NiV) in Kerala, India, captured global attention as it led to two reported fatalities and hospitalizations of several locals experiencing neurological and respiratory distress [1]. Despite the infrequent sporadic outbreaks of NiV [2], its fatality rates can exceed 90% [3]. This virus was first identified during an outbreak in Malaysia in 1998, causing more than 265 encephalitis cases and 105 deaths [4]. From 2001 to 2019, it was reported sporadically in South and Southeast Asian countries, with almost 400 cases and 47 deaths [3].

NiV or *Henipavirus nipahense*, belongs to the *Paramyxoviridae* family. Its viral genome from 3’-5’ consists of six genes, *viz*., nucleocapsid (N), phosphoprotein (P), matrix (M), fusion glycoprotein (F), attachment glycoprotein (G), and long polymerase (L) [5]. This virus is a zoonotic pathogen naturally hosted by fruit bats of the *Pteropus* genus [6]. It can also infect other mammals, including humans, due to its ability to interact with the highly conserved mammalian ephrin receptor [5].

Pigs are the most common veterinary vehicles of NiV to humans. It was linked to the first outbreak in Malaysia and the transboundary movement of the virus in Southeast Asia [4]. During the 1999 outbreak in Malaysia, approximately 45% of the country’s pig population or more than 1.1 million pigs were culled [7], incurring significant economic loss and long-term damage to the pig industry. In addition to pig-related transmission, there was also a reported NiV outbreak associated with horse meat consumption in the Philippines in 2014. This incident involved nine deaths, with a case fatality rate of almost 53% [8].

While NiV holds significance as an emerging disease with pandemic potential, there are currently no approved therapeutics or vaccines for use in humans or any other intermediate hosts of the virus [9]. Owing to the lethal nature of NiV, developing a safe live-attenuated vaccine with no risk of reversion is challenging [7]. Efforts to develop an NiV vaccine have focused on using viral vectors and subunit-based designs [10]. However, despite encouraging results from multiple experimental viral-vectored vaccine candidates in challenge studies [7, 11], none have yet reached the market. In developing vaccine candidates, an epitope-based subunit design was recommended as it is safer than traditional live-attenuated designs [12–14].

Epitope-based subunit vaccines focus on essential antigenic elements of the pathogen [15, 16]. Many of these types, especially for cancers, have progressed rapidly from pre-clinical trials to clinical trials [17]. In the ongoing effort to combat the spread of NiV, a growing number of studies have proposed epitope-based subunit vaccine designs against the virus for experimental validation [18–24]. However, these designs were specifically for human immunizations. Given the zoonotic nature of NiV, exploring strategies that can also target its intermediate hosts is deemed essential.

The concept of ‘One Health Vaccinology’ [25] introduces a transformative approach wherein synergies between human and veterinary immunology are recognized and leveraged for the development of vaccines. In this paradigm, vaccinations are targeted at animals to prevent human diseases. However, existing biological parallels between humans and animals, including host-pathogen interactions, structure and composition of the immune systems, and even vaccine development pipelines from design to evaluation, suggest that a cross-species vaccine can be feasible [26]. Currently, no licensed vaccines are available for use across humans and veterinary animals. However, similar initiatives have been undertaken for the zoonotic Rift Valley Fever virus (RVFV) [27]. This replication-defective chimpanzee adenovirus vaccine encoding RVFV envelope glycoproteins was able to elicit high-titer neutralizing antibodies in sheep, goat, and cattle [27] and has already established safety for use in humans [28]. This achievement underscores the potential use of cross-species vaccines in addressing other zoonotic pathogens, like NiV [26].

The present study introduces the first multi-epitope subunit vaccine designed for cross-species immunization against NiV using immunoinformatics and molecular modeling. Immunoinformatics, through its rich computational tools and resources, was used to map potential immunogenic determinants (epitopes) in NiV protein sequences that elicit cellular and humoral immune responses in humans, swine, and horses. These epitopes were joined by peptide linkers forming a vaccine construct and were added with an adjuvant to boost its immunogenicity. The designed vaccine construct was evaluated using multiple tools to analyze the physicochemical properties and predict its immunogenicity to hosts upon immunization. Molecular modeling analyses predicted the three-dimensional structure of the vaccine construct where docking and dynamics simulations assessed its interaction with host immune receptors. This study also compared the vaccine construct’s safety, stability, and immunogenicity to six documented vaccine designs for NiV in the literature.

## Materials and methods

**Fig 1:**
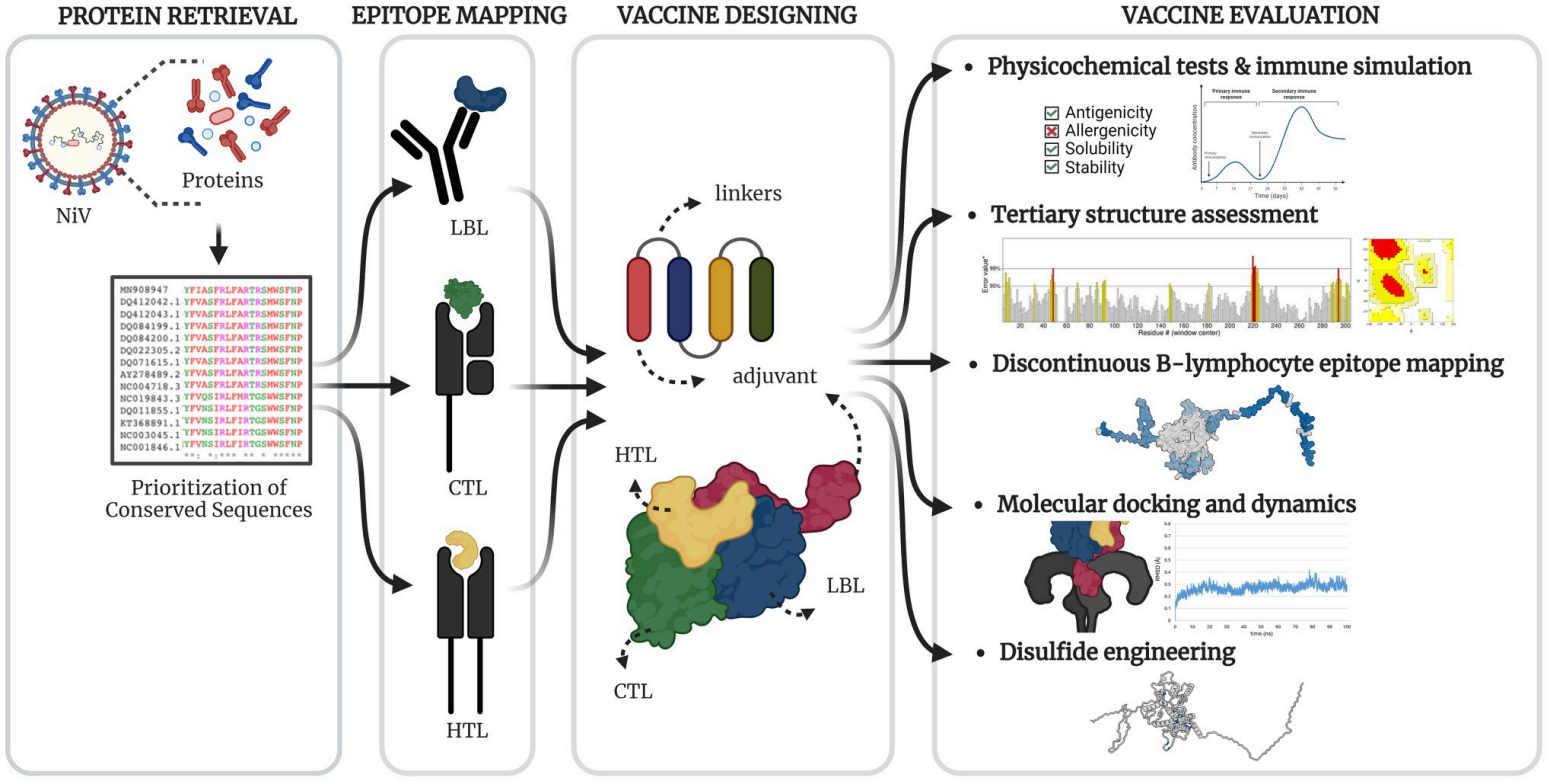
General methodology employed in the study. The multi-epitope subunit vaccine design pipeline employed in this study was categorized into four groups, as delineated by boxes: protein retrieval, epitope mapping, vaccine design, and vaccine evaluation. Figure created using BioRender.com.

The entire pipeline was carried out using *in silico* methods. The summary of the methodology is presented in Fig 1. All databases and servers were accessed from September 2023 to February 2024.

### Protein retrieval

Full-length sequences of the nine *Henipavirus nipahense* (taxid:3052225) reference proteins were retrieved from NCBI [29] (https://www.ncbi.nlm.nih.gov). Isoforms of these proteins from the same virus were retrieved using BLASTP suite [30] (https://blast.ncbi.nlm.nih.gov/Blast.cgi), filtering with a query cover of >80%. Models (XM/XP), non-redundant RefSeq proteins (WP), and uncultured/environmental sample sequences were excluded from the search. Retrieved sequences were aligned using Clustal Omega [31] (https://www.ebi.ac.uk/Tools/msa/clustalo) and the resulting alignment was uploaded to the Protein Variability Server (PVS) [32] (http://imed.med.ucm.es/PVS) to identify highly conserved fragments. Shannon variability entropy (H) ≥1.0 was set for highly variable residues. Contiguous conserved residues were selected for epitope prediction. Fragments of ≥16, ≥9, and ≥15 residues were retrieved for linear B-lymphocyte (LBL) epitope, cytotoxic T-lymphocyte (CTL) epitope, and helper T-lymphocyte (HTL) epitope predictions, respectively.

### Epitope mapping

#### LBL epitope prediction

Fragments of ≥16 residues were submitted to BepiPred 3.0 server [33] (https://services.healthtech.dtu.dk/services/BepiPred-3.0) for initial prediction, using default parameters. Epitopes predicted by BepiPred 3.0, were submitted to the SVMTrip server [34] (http://sysbio.unl.edu/SVMTriP/prediction.php) and ABCPred server [35] (https://webs.iiitd.edu.in/raghava/abcpred) for cross-validation. A threshold of 0.85 was used for ABCPred while parameters from SVMTrip were kept at default. Epitopes validated either by SVMTrip or ABCPred were submitted to the LBtope server [36] (https://webs.iiitd.edu.in/raghava/lbtope) to assess the confidence of the prediction. Only epitopes with a probability ≥60% were considered for subsequent analyses.

#### CTL epitope prediction

Twenty-five reference human leukocyte antigens (HLAs) [37] and 45 commonly used swine leukocyte antigens (SLAs) [38–41] were used as receptors for CTL epitope prediction. Fragments of ≥9 residues were submitted to NetMHCcons 1.1 [42] (https://services.healthtech.dtu.dk/services/NetMHCcons-1.1). Epitopes with a percentile rank (PR) ≤0.5 to at least one HLA and SLA were selected. These epitopes were submitted into NetCTLpan 1.1 [43] (https://services.healthtech.dtu.dk/services/NetCTLpan-1.1). Epitopes with a PR ≤1 to at least one HLA and SLA were selected. Class I immunogenicity prediction [44] (http://tools.iedb.org/immunogenicity) further screened the epitopes using a score >0.

#### HTL epitope prediction

Twenty-seven reference HLAs [45] and 43 HLAs that are known SLA-equivalents [46] were used as receptors for HTL epitope prediction. Fragments of ≥15 residues were submitted to the Immune Epitope Database Analysis (IEDB) MHC II binding prediction [47] (http://tools.iedb.org/MHCii/). Epitopes with a PR <10 were selected. These epitopes were submitted to NetMHCIIpan 4.3 [48] (https://services.healthtech.dtu.dk/services/NetMHCIIpan-4.3). Epitopes with a PR <1 were selected. CD4 T-cell immunogenicity prediction tool [49] (http://tools.iedb.org/CD4episcore) further screened the epitopes using a combined score ≥40. Epitopes that were predicted to positively induce at least one of the cytokines: tumor necrosis factor (TNF) *α* in TNFepitope [50] (https://webs.iiitd.edu.in/raghava/tnfepitope), interleukin-6 (IL6) in IL-6Pred [51] (https://webs.iiitd.edu.in/raghava/il6pred), and interferon (IFN) *γ* in IFNepitope [52] (https://webs.iiitd.edu.in/raghava/ifnepitope), were retrieved.

#### Physicochemical tests

Potential for antigenicity in Vaxijen 2.0 [53] (https://www.ddg-pharmfac.net/vaxijen/VaxiJen/VaxiJen.html), non-allergenicity in AllerTOP v2.0 [54] (https://www.ddg-pharmfac.net/AllerTOP/feedback.py), and nontoxicity in ToxinPred [55] (https://webs.iiitd.edu.in/raghava/toxinpred/multipletest.php), further screened the LBL, CTL, and HTL epitopes.

#### Molecular docking and dynamics of epitopes to MHC molecules

Sequences of the five most prevalent MHC I and MHC II molecules found in swine [46], human [56], and equine [57] populations were retrieved from the Immuno Polymorphism Database (IPD) [58] (https://www.ebi.ac.uk/ipd). SWISS-MODEL server homology modeling pipeline [59] (https://swissmodel.expasy.org) was employed for structure prediction. Subsequent energy minimizations were performed using Chimera 1.17.1 [60], applying 100 steps of steepest descent. Structure refinements were performed using the Refine2 service [61] of GalaxyWEB (http://galaxy.seoklab.org/refine). The quality of the predicted structures was determined using ERRAT [62] and PROCHECK [63] services in SAVES v6.0 (https://saves.mbi.ucla.edu), and the models with the highest scores from the quality assessment servers were selected as the representative models of MHC molecules.

Crystal structures of MHC molecules with docked peptides were retrieved in RCSB Protein Data Bank [64] (https://www.rcsb.org) and were used as positive controls. Epitopes were docked to the representative models of MHC molecules using HPepDock 2.0 [65] (http://huanglab.phys.hust.edu.cn/hpepdock). The ten best complexes were retrieved from the server. Using the hclust function [66] in RStudio [67], the epitopes with binding regions similar to the controls were screened using a 20-height threshold. Binding affinity analyses were performed using the Molecular Mechanics/Generalized Born Surface Area (MM/GBSA) service [68] available in HawkDock (http://cadd.zju.edu.cn/hawkdock). Epitopes with the lowest energy per complex were selected as the vaccine construct’s final CTL and HTL components.

Molecular dynamics of the epitope complexes were conducted using CHARMM36 force field [69] on GROMACS 2023.2. [70]. System energy minimizations were performed to reach a maximum of 50,000 steps of steepest descent. Systems were set to equilibrium at a temperature of 300K and a pressure of 1 bar. The gmxMMGBSA tool [71] was used to calculate the binding energies of the complexes and determine the per-residue energy decomposition at 8 Å.

### Vaccine designing

The predicted LBL epitopes were connected using the KK linker, CTL epitopes using the AAY linker, and HTL epitopes using the GPGPG linker. The three epitope groups were fused using the HEYGAEALERAG linker. This multi-epitope construct was attached to seven different adjuvants: resuscitation-promoting factor E (RpfE), flagellin, 50S ribosomal protein L7/L12 (50SrpL7/L12), phenol-soluble modulin *α*4 (psm*α*4), cholera toxin B (CTB), truncated human heat shock protein 70 (tHsp70), and truncated human *β*-defensin 3 (th*β*d3), using the EAAAK linker. The host immune responses induced by these seven constructs were predicted using C-Immsim [72] (https://kraken.iac.rm.cnr.it/C-IMMSIM). Three injections of 1000 vaccine particles were administered at 1–84-168 time-steps and the simulation was run for 300 time-steps. The graphs of antibody titers, interferon-*γ* concentration, B-lymphocyte populations, and cytotoxic (T_C_) and helper lymphocyte (T_H_) populations induced by the constructs were compared. Aside from the immune simulation results, several criteria were also applied to select the final vaccine construct: (1) ≥0.4 antigenicity score in VaxiJen v2.0, (2) allergenic potential in AllerTOP v2.0, (3) solubility upon overexpression in *Escherichia coli* in SCRATCH SolPro [73] (https://scratch.proteomics.ics.uci.edu), and (4) stability in Expasy Protparam [74] (https://web.expasy.org/protparam).

### Vaccine evaluation

#### Immune simulation and physicochemical tests

Computationally-designed multi-epitope subunit NiV vaccine NiV sequences (2020–2023) were retrieved from the literature. Host immune response simulations were generated using the C-ImmSim server by applying the abovementioned parameters. Graphs of antibody titers, interferon-*γ* concentration, B-lymphocyte, T_C_, and T_H_ populations induced by the constructs were generated. Additionally, their antigenicity, allergenicity, solubility, and stability were determined. The immune simulation profile and physicochemical properties of the constructs were compared to the construct of the study.

#### Tertiary structure assessment

The tertiary structure of the vaccine construct in this study was predicted using three servers: (1) ColabFold v1.5.2-patch [75] available in AlphaFold v2 [76] (https://colab.research.google.com/github/sokrypton/ColabFold/blob/main/AlphaFold2.ipynb), using pdb100 for detection of templates; (2) Deep learning-based Iterative Threading ASSEmbly Refinement (D-I-TASSER) server [77] (https://zhanggroup.org/D-I-TASSER); and (3) RoseTTAFold [78] available in Robetta server (https://robetta.bakerlab.org/submit.php). The predicted models were energy-minimized using Chimera 1.17.1 at 100 steps of steepest descent. Further refinement was performed using the GalaxyWEB Refine service. The quality of the predicted structures was assessed using ProSA-web server [79] (https://prosa.services.came.sbg.ac.at/prosa.php/), ERRAT, and PROCHECK services. The model with the highest scores on these quality servers was used as the representative model of the vaccine construct.

#### Conformational B-lymphocyte epitope prediction

The conformational B-lymphocyte epitopes within the vaccine construct were predicted using the Ellipro tool [80] (http://tools.iedb.org/ellipro/), applying its default parameters.

#### Molecular docking and dynamics analyses of vaccine construct to TLR

The human toll-like receptor (TLR) 4 crystal structure was downloaded from RCSB. Peptides bound to the structure, water molecules, and unwanted atoms were removed using Pymol v2 [81]. Missing residues within the structure were filled using Modeller [82] (https://salilab.org/modeller). The representative model of the vaccine construct was docked to the TLR4 structure using ClusPro 2.0 [83] (https://cluspro.bu.edu/login.php). The complex’s overall binding affinity and per-residue energy decomposition were determined using HawkDock. TLR4 residues significant for the agonist (lipopolysaccharide) binding were used to identify the representative model of the docked complex. The stability of the complex was analyzed using SIRAH force-field [84] on GROMACS 2023.2 [70]. System energy minimization was performed to reach the maximum of 50,000 steps of steepest descent. The system was set to equilibrium at a temperature of 300K and a pressure of 1 bar. Stable complexes, with constant RMSD trajectory at approximately 20–30 ns after equilibration [85, 86], were noted.

#### Disulfide engineering

Disulfide by Design 2.0 [87] (http://cptweb.cpt.wayne.edu/DbD2/) was used to identify potential residue pairs within the vaccine construct that could be modified to cysteine for increased stability. Default parameters were applied in the analysis and a bond energy threshold of ≤ 2.0 kcal/mol was used to screen residue pairs.

#### Codon optimization and *in silico* cloning

VectorBuilder (https://en.vectorbuilder.com/tool/codon-optimization.html) was used to generate the vaccine construct’s optimized codon sequence. *Escherichia coli* K-12 substrain MG1655 was used as the expression system. BamHI and XhoI restriction enzymes were added to the N-terminal and C-terminal sites of the sequence, respectively. This sequence was cloned into the sample vector, pET-28a(+), using the restriction cloning module of the SnapGene tool (http://www.snapgene.com).

## Results and discussion

### Protein retrieval

The present study used all known NiV proteins as epitope sources for vaccine designing. Previous multi-epitope subunit vaccine designs for NiV only used the exposed viral structural proteins, G and F, for epitope mapping due to their interaction with host cell receptors [20, 22]. Although nonstructural proteins may not be readily accessible for interaction with lymphocyte receptors during their synthesis within virus-infected cells, their release upon cell lysis enables their binding to lymphocyte receptors which can subsequently initiate an immune response cascade. Several studies that designed multi-epitope subunit vaccines for NiV have considered the potential role of nonstructural proteins in activating immune responses [18, 19, 21, 23, 24], an approach that has also been adopted in this study.

The reference sequences of the three structural and six nonstructural NiV proteins were downloaded from NCBI: N (NP_112021.1), P (NP_112022.1), M (ID:NP_112025.1), F (ID:NP_112026.1), G (ID:NP_112027.1), L (ID:NP_112028.1), V (NP_112023.1), W (ID:APT69630.1), and C (ID:NP_112024.1). These sequences were used as input data to retrieve all the NiV protein variants for subsequent conservation analyses. The conservation of epitopes within species variants is a highly advantageous feature of a vaccine as it maximizes coverage, making the vaccine more universally effective across diverse populations [15]. The protein variants were used in this study to identify conserved regions within sequences.

Sixty-seven peptides with ≥9 conserved residues were retrieved for CTL epitope prediction. Major histocompatibility complex I (MHC I) receptors generally bind to peptides with 9 amino acids [88]. Peptides shorter than this length may not provide sufficient interaction for effective binding and presentation, potentially affecting the efficacy of the immune response. In HTL epitope prediction, longer peptides, approximately 15 amino acids in length, can be accommodated by MHC II receptors due to their open binding groove conformation [52, 88]. In this study, 55 peptides with ≥15 conserved residues were obtained. While LBL epitope prediction allows for a more dynamic range in peptide length, some servers have specific length requirements. Fifty-four peptides with ≥16 conserved residues were retrieved for LBL epitope prediction.

### Epitope mapping

This study aims to design a vaccine that can effectively activate B-lymphocytes. Using the peptides with ≥16 conserved residues, the first B-lymphocyte epitope prediction server, BepiPred 3.0, identified 222 potential LBL epitopes. These epitopes were validated using SVMTrip and ABCPred. The use of multiple servers for validation increases the confidence of the prediction [41], and the use of SVMTrip and ABCPred servers for validation follows the B-lymphocyte epitope prediction pipeline of DeepVacPred [89], a framework that applies deep neural network architecture for designing multi-epitope subunit vaccines. Meanwhile, the use of BepiPred 3.0 as the initial prediction server is justified by its superior accuracy compared to other LBL epitope prediction servers in a recent benchmark study [90]. From the 222 predicted B-lymphocyte epitopes by BepiPred 3.0, 163 16-mer epitopes were validated by ABCPred while two 20-mer epitopes were validated by SVMTrip. From the 165 validated LBL epitopes, the LBtope server identified 34 epitopes with a 60% correct prediction rate, which was the recommended threshold of the server. LBtope is known for using validated non-linear epitopes for predictions instead of random peptides [36].

The epitopes were tested for their antigenicity and safety properties. Antigenicity tests were conducted using Vaxijen 2.0, a sequence-based tool that classifies antigens based on physicochemical properties. Protective antigens are prioritized in vaccine designs because of their ability to mount immune memory [53]. Aside from being antigenic, epitope components of the vaccine should be safe. AllerTOP and ToxinPred are servers that predict the epitopes’ potential allergenicity and toxicity. These servers analyze the physicochemical properties of epitopes and their similarities to known allergens and toxic motifs. A vaccine with epitopes that mimic proteins or peptides that are known allergens or toxins should be avoided, as it could lead to adverse reactions in vaccinated individuals [54, 55].

Only ten LBL epitopes satisfied the antigenicity, allergenicity, and toxicity thresholds. These epitopes were selected as the final LBL epitope components of the vaccine construct. Table 1 shows the scores of these LBL epitopes across the servers used for physicochemical tests and their positions in their respective protein sources.

**Table 1:** Predicted LBL epitopes, their positions in protein sources, and scores from prediction and physicochemical tests.

Epitopes	Protein source^a^	% Correct Prediction	Antigenicity Score^b^	Allergenicity	Toxicity Score^c^
KYKIYTPGANERKYNN	M_46–61_	74.00	0.46	Non-allergen	-1.33
YGRSSIQQPSIKDRTK	PVW_28–43_	77.81	0.82	Non-allergen	-1.62
NTRDWAEGSDDIQLDP	PVW_94–109_	62.76	0.93	Non-allergen	-1.12
CLVSDAKVLSYAPEIA	PVW_149–164_	62.94	0.62	Non-allergen	-0.91
KENSFINSQQGKDAQP	PVW_347–362_	71.38	0.92	Non-allergen	-0.91
YRGIEGSRSPDKTEIT	PVW_364–380_	70.21	0.91	Non-allergen	-0.86
RSPDKTEITSDAVQTA	PVW_370–387_	72.20	0.40	Non-allergen	-1.06
HVRGSPPYQEGKSVNA	P_430–445_	70.74	0.97	Non-allergen	-0.19
TPMPKSRGIPIKKGHR	V_394–409_	63.28	1.53	Non-allergen	-1.08
LGRRVVQPGMFEDHPP	W_421–436_	79.39	0.64	Non-allergen	-0.57

^a^position relative to the reference sequence;

^b^≥0.4 score designates a peptide antigenic;

^c^≤0 score designates a peptide non-toxic.

In addition to B-lymphocytes, activation of T-lymphocytes, comprising both cytotoxic (CTL) and helper (HTL) subsets, is also an important characteristic of a vaccine. Memory T-lymphocytes can contribute to the establishment of long-lasting immunity against recurrent pathogen infections. However, successful CTL and HTL induction requires efficient antigen processing and presentation. Antigen processing involves the breakdown of antigens, such as proteins or peptides into smaller fragments that can be recognized by immune cells. These fragments are subsequently loaded into MHC molecules for presentation to T-lymphocytes.

CTL activation involves T-cell receptor (TCR) recognition of peptide-bound MHC I. From the 67 peptides with ≥9 conserved residues, this study evaluated 4,343 9-mer peptides for their potential to activate CTLs. The first consideration is that the internal residues of the peptide should not match the cleavage sites of the cytosolic proteasome. CTL responses may diminish when epitopes are destroyed by cytosolic proteasomes [91]. Second, it is necessary to consider the efficiency of the peptides to be transported by the transporter associated with antigen processing (TAP) complex into the endoplasmic reticulum (ER). This is approximated by evaluating the binding affinity of peptides to TAP [92–94]. Within the ER, peptides bind to MHC I and are transferred to the cell surface for presentation. These successive steps from a protein or a long peptide precursor to a 9-residue peptide binding to MHC I molecules collectively constitute antigen processing. These steps are integrated into the overall prediction results of the NetCTLpan server.

Biologically, for a peptide to be a good CTL epitope, a favorable affinity to MHC I molecules is insufficient [44]. The peptide complexed with MHC I (pMHC I) needs to be recognized by CD8+ precursor CTL receptors to carry out effector functions. Class I immunogenicity prediction distinguishes pMHC I molecules that are effectively recognized by T-lymphocytes (immunogenic), and those for which no recognizing T-lymphocytes exist (non-immunogenic). This discrimination is achieved by analyzing trends within a substantial set of immunogenic and non-immunogenic pMHCs [44].

Considering antigenicity, processing, binding affinity to human MHC I molecules, and immunogenicity, 109 CTL epitopes were screened. However, only 59 of these epitopes passed evaluations for allergenicity and toxicity. Furthermore, after considering the processing and binding affinity evaluations using swine MHC I molecules, only 17 CTL epitopes remained. Using swine MHC I molecules ensured the ability of the peptide to induce cross-species protection. Table 2 presents the CTL epitope scores across the servers used for physicochemical tests and their positions in their respective protein sources.

**Table 2:** Predicted CTL epitopes, their positions in protein sources, and scores from physicochemical tests.

Epitopes	Protein source^a^	Antigenicity Score^b^	Allergenicity	Toxicity Score^c^	No. of strong binding receptors^d^	Immunogenicity Score^e^
AQITAGVAL	F_126–134_	0.80	Non-allergen	-1.23	1 HLA; 2 SLA	0.21
QITAGVALY	F_127–135_	0.66	Non-allergen	-1.22	2 HLA; 1 SLA	0.15
YLSDLLFVF	F_206–214_	0.69	Non-allergen	-1.23	3 HLA; 7 SLA	0.07
ITIPANIGL	G_118–126_	1.11	Non-allergen	-1.00	1 HLA; 2 SLA	0.20
AMDEGYFAY	G_223–231_	0.72	Non-allergen	-0.83	3 HLA; 15 SLA	0.26
YMYLICYGF	M_62–70_	1.13	Non-allergen	-0.63	3 HLA; 6 SLA	0.08
KFAPGGYPL	N_321–329_	1.01	Non-allergen	-0.48	1 HLA; 2 SLA	0.06
SFLDYHTEF	L_677–685_	0.94	Non-allergen	-0.86	4 HLA; 2 SLA	0.13
ESMAIFAER	L_733–741_	0.80	Non-allergen	-1.44	3 HLA; 1 SLA	0.31
YGLPGFFNW	L_746–754_	1.13	Non-allergen	-0.59	3 HLA; 3 SLA	0.22
WTIATIPFL	L_806–814_	1.08	Non-allergen	-0.87	5 HLA; 3 SLA	0.30
TIATIPFLF	L_807–815_	0.93	Non-allergen	-0.77	2 HLA; 4 SLA	0.26
IPFLFLSAY	L_811–819_	0.90	Non-allergen	-0.69	1 HLA; 2 SLA	0.01
FLDWASDPY	L_1050–1058_	0.49	Non-allergen	-0.70	2 HLA; 9 SLA	0.12
NTMYGWFFV	L_1243–1251_	0.63	Non-allergen	-0.32	4 HLA; 8 SLA	0.35
FPLWSTEEL	L_1483–1491_	0.77	Non-allergen	-0.91	4 HLA; 1 SLA	0.24
IAYTPGFPI	L_1977–1985_	0.53	Non-allergen	-1.04	1 HLA; 9 SLA	0.15

^a^position relative to the reference sequence;

^b^≥0.4 designates a peptide antigenic;

^c^≤0 designates a peptide non-toxic;

^d^in humans (HLA) and swine (SLA);

^e^positive score indicates a likelihood of T-lymphocyte recognition in complex with MHC I.

On the other hand, antigen processing for MHC II presentation occurs through the internalization of viral or vaccine particles by antigen-presenting cells and degradation into peptides in the endocytic processing pathway compartments [95, 96]. Antigens enter cells through endosomes and fuse with lysosomes containing proteases and membrane-bound MHC II molecules. The enzymatic activity of proteases, coupled with acidic environment is responsible for the cleavage of antigens into peptides [96]. Therefore, for MHC II presentation, prediction of cytosolic proteasomal cleavage is not required. TAP transport efficiency is also not required because peptides do not need to enter the ER for MHC binding. MHC II molecules involved in the presentation are already present in the endosomal-lysosomal compartments.

Antigens in the compartments are degraded into oligopeptides that are usually 13–25 amino acids in length [97]; however, peptides that are 15 amino acids are commonly observed [52]. In this study, 4,039 15-mer peptides were derived from the 55 peptides with ≥15 conserved residues. The HTL epitope processing efficiency is conducted using antigenicity tests and binding affinity analysis to human MHC II molecules. These analyses were able to screen 1,169 epitopes. Only 635 of these epitopes passed evaluations for allergenicity and toxicity. Additionally, only 24 epitopes have strong binding affinity to swine MHC II molecules. Table 3 presents the scores of these HTL epitopes across the servers used for physicochemical tests, and their positions from their protein sources.

**Table 3:** Predicted HTL epitopes, their positions from protein sources, and scores from physicochemical tests.

Epitopes	Protein source^a^	Antigenicity Score^b^	Allergenicity	Toxicity Score^c^	No. of strong binding receptors^d^	Immunogenicity Score^e^
DIVIKMIPNVSNMSQ	F_56–70_	0.58	Non-allergen	-0.76	1 HLA; 6 SLA	35.52
DNSEWISIVPNFILV	F_304–318_	0.74	Non-allergen	-0.84	1 HLA; 12 SLA	30.40
SEWISIVPNFILVRN	F_306–320_	0.58	Non-allergen	-1.07	1 HLA; 14 SLA	27.58
EWISIVPNFILVRNT	F_307–321_	0.83	Non-allergen	-1.03	1 HLA; 5 SLA	30.34
VYNDAFLIDRINWIS	G_507–521_	0.57	Non-allergen	-1.12	1 HLA; 4 SLA	40.11
KRKKIRTIAAYPLGV	M_82–96_	0.41	Non-allergen	-0.95	6 HLA; 42 SLA	34.98
RKKIRTIAAYPLGVG	M_83–97_	0.86	Non-allergen	-1.03	7 HLA; 43 SLA	35.05
TMLEFRRNNAIAFNL	M_192–206_	1.28	Non-allergen	-0.75	1 HLA; 4 SLA	26.93
MLEFRRNNAIAFNLL	M_193–207_	1.15	Non-allergen	-1.14	2 HLA; 7 SLA	28.31
LHIKINGVISKRLFA	M_276–290_	0.57	Non-allergen	-1.13	1 HLA; 7 SLA	37.01
QSLFSFDNVKNFRDG	P_606–620_	0.57	Non-allergen	-1.27	2 HLA; 14 SLA	37.19
SLFSFDNVKNFRDGS	P_607–621_	0.69	Non-allergen	-1.40	2 HLA; 5 SLA	39.38
PNLICIFKSDKTGKK	L_202–226_	0.91	Non-allergen	-0.42	1 HLA; 9 SLA	34.74
GITSVIFKIKNSQSK	L_2046–2060_	0.55	Non-allergen	-1.35	1 HLA; 13 SLA	40.12
ITSVIFKIKNSQSKQ	L_2047–2061_	0.52	Non-allergen	-1.16	2 HLA; 15 SLA	40.73
SVIFKIKNSQSKQFH	L_2049–2063_	0.48	Non-allergen	-0.50	1 HLA; 12 SLA	37.89
KKVIVYSLIKFKDTK	L_2162–2176_	0.48	Non-allergen	-1.15	1 HLA; 6 SLA	39.17
IRRKVLILDFRSKLM	L_2187–2201_	1.20	Non-allergen	-1.50	1 HLA; 3 SLA	39.94
RRKVLILDFRSKLMT	L_2188–2202_	1.13	Non-allergen	-1.86	1 HLA; 3 SLA	42.31
RKVLILDFRSKLMTK	L_2189–2203_	1.05	Non-allergen	-2.05	2 HLA; 8 SLA	41.71
KVLILDFRSKLMTKT	L_2190–2204_	0.88	Non-allergen	-2.11	2 HLA; 8 SLA	38.50
MASILLTLFRRTKKK	C_2–16_	0.55	Non-allergen	-2.03	3 HLA; 1 SLA	36.47
ASILLTLFRRTKKKY	C_3–17_	0.50	Non-allergen	-1.94	2 HLA; 1 SLA	38.11
SILLTLFRRTKKKYR	C_4–18_	0.55	Non-allergen	-1.93	1 HLA; 1 SLA	33.87

^a^position relative to the reference sequence;

^b^≥0.4 designates a peptide antigenic;

^c^≤0 designates a peptide non-toxic;

^d^in humans (HLA) and swine (SLA);

^e^≤50 score indicates likelihood of T-lymphocyte recognition in complex with MHC II.

The HTL epitopes were evaluated for their ability to induce cytokines. Cytokine induction is an integral component of HTL response. When HTL epitopes bind to MHC II and are recognized by TCRs, they trigger intracellular signaling pathways that release cytokines. This study screened only those HTL epitopes capable of inducing at least one cytokine significant for NiV control. Studies of NiV infection in experimental animal models show that cytokines IL1*α*, IL1*β*, IL6, IL8, TNF*α*, IFN*γ*, granulocyte-colony stimulating factor, and C-X-C motif chemokine 10 are induced in infected organisms [5, 98]. Three of these cytokines, TNF*α*, IL6, and IFN*γ*, have available prediction servers; hence, were the only cytokine predictions included in the analysis. Only 20 of the 24 HTL epitopes were predicted to induce at least one of these cytokines (See Table 4).

**Table 4:** HTL epitopes associated with at least one cytokine induction.

Epitopes	IFN*γ* Score^a^	IL4 Score^b^	IL10 Score^c^
SEWISIVPNFILVRN	- (0.00)	+ (1.05)	+ (0.32)
EWISIVPNFILVRNT	- (0.00)	+ (1.06)	- (0.22)
VYNDAFLIDRINWIS	+ (0.24)	+ (0.26)	+ (0.48)
KRKKIRTIAAYPLGV	- (0.00)	- (0.02)	+ (0.66)
RKKIRTIAAYPLGVG	- (0.00)	- (0.12)	+ (0.58)
TMLEFRRNNAIAFNL	- (-0.75)	+ (0.32)	- (-0.03)
MLEFRRNNAIAFNLL	- (-0.96)	+ (0.21)	- (0.24)
LHIKINGVISKRLFA	- (-0.05)	+ (0.44)	+ (0.38)
QSLFSFDNVKNFRDG	- (-0.54)	+ (0.27)	+ (0.45)
GITSVIFKIKNSQSK	+ (0.25)	+ (0.47)	+ (0.35)
SVIFKIKNSQSKQFH	- (-0.30)	+ (0.37)	+ (0.75)
ITSVIFKIKNSQSKQ	- (-0.27)	+ (1.16)	+ (0.33)
KKVIVYSLIKFKDTK	- (0.00)	+ (1.33)	+ (0.94)
IRRKVLILDFRSKLM	+ (0.10)	+ (0.36)	- (0.13)
RRKVLILDFRSKLMT	+ (0.14)	+ 90.31)	- (0.24)
RKVLILDFRSKLMTK	+ (0.04)	+ (1.37)	- (0.17)
KVLILDFRSKLMTKT	- (-0.17)	+ (0.37)	- (0.01)
MASILLTLFRRTKKK	+ (0.01)	- (0.08)	- (0.09)
ASILLTLFRRTKKKY	+ (0.01)	- (-1.43)	- (-0.15)
SILLTLFRRTKKKYR	- (0.00)	+ (0.90)	- (0.11)

^a^positive score designates a peptide an IFN*γ* inducer;

^b^≥0.2 score designates a peptide an IL4 inducer;

^c^^b^≥0.3 score designates a peptide an IL10 inducer.

#### Molecular docking and dynamics of epitopes to MHC molecules

The binding of peptides to MHC molecules is the most significant process in antigen presentation [43], and is the major contributor to immunodominance in T-lymphocyte responses [99]. This study conducted molecular docking and dynamics analyses to ensure that the predicted epitopes bind efficiently to MHC molecules. Molecular docking explores the fitting of the epitopes to the binding sites of MHC molecules; whereas, dynamic analyses delve into the temporal aspects of the interactions, assessing the stability of the complexes over time [100–103].

Fifteen MHC I and 15 MHC II sequences were retrieved for docking analysis (See S1 Fig). These MHCs represent the prevalent MHC I and II molecules in human [45, 56], swine [46], and equine [57, 104] populations. Their structures were generated using the homology feature of SWISS-MODEL. These structures were further energy-minimized and refined to correct errors, relieve steric clashes, and enhance accuracy, ensuring physical feasibility and alignment with experimental data. Most of the generated structures achieved high-quality scores on quality assessments (See S1 Table), reflecting their reliability for biological insights. The initially predicted CTL and HTL epitopes were docked with MHC I and MHC II, respectively. Among the predicted epitopes, only a subset shared a binding conformation similar to control peptides used in the analysis (See S2 Fig). The selected control peptides (See S2 Table) from other pathogenic sources were already known to interact efficiently with MHC molecules under laboratory conditions. The similarity of binding conformations to these peptides suggests the potential for a similar MHC binding efficacy. However, conformation alignment to the control peptides is insufficient; the predicted epitopes should also exhibit a strong affinity to MHC molecules [105, 106]. The vaccine construct’s final CTL and HTL components were determined by selecting the epitopes from complexes with the lowest energy for each MHC I and MHC II molecule (See S3 Fig). This process was designed to selectively incorporate epitopes that demonstrated highly favorable interactions with MHC molecules, limiting the number of epitopes in the construct. Table 5 presents the final list of CTL and HTL epitope components of the vaccine and MHCs, which demonstrated superior binding affinity.

**Table 5:** Final list of CTL and HTL epitope components of the vaccine and the MHCs in which it demonstrated superior binding affinities.

Epitopes	Strong binding MHC/s
TIATIPFLF	HLA-A*02:01, SLA-2*02:01, Eqca-2*00201, Eqca-2*003:01
ESMAIFAER	HLA-A*02:02, SLA-2*04:01
ITIPANIGL	HLA-A*02:03
AQITAGVAL	HLA-A*02:06
IAYTPGFPI	HLA-A*68:02
YMYLICYGF	SLA-1*04:01, Eqca-1*003:01
IPFLFLSAY	SLA-1*08:01
FLDWASDPY	SLA-3*04:01
SFLDYHTEF	Eqca-2*004:01
QITAGVALY	Eqca-N*006:01
EWISIVPNFILVRNT	HLA-DP(A1*01:03-B1*04:02)
RKKIRTIAAYPLGVG	HLA-DQ(A1*03:01-B1*03:02), SLA-DQ(A*02:01-B1*02:01)
	Eqca-DQ(A1*002:01-B1*002:01)
SVIFKIKNSQSKQFH	HLA-DQ(A1*05:01-B1*03:01), Eqca-DR(A*001:01-B1*002:01)
RKVLILDFRSKLMTK	SLA-DQ(A*01:01-B1*07:01), Eqca-DR(A*001:01-B1*001:01)
SEWISIVPNFILVRN	SLA-DR(A*01:01-B1*04:01)
RRKVLILDFRSKLMT	SLA-DR(A*01:01-B1*06:01), Eqca-DR(A*001:01-B2*001:01)
MASILLTLFRRTKKK	SLA-DR(A*01:01-B1*10:01)
QSLFSFDNVKNFRDG	Eqca-DQ(A1*001:01-B1*001:01)

**Fig 2:**
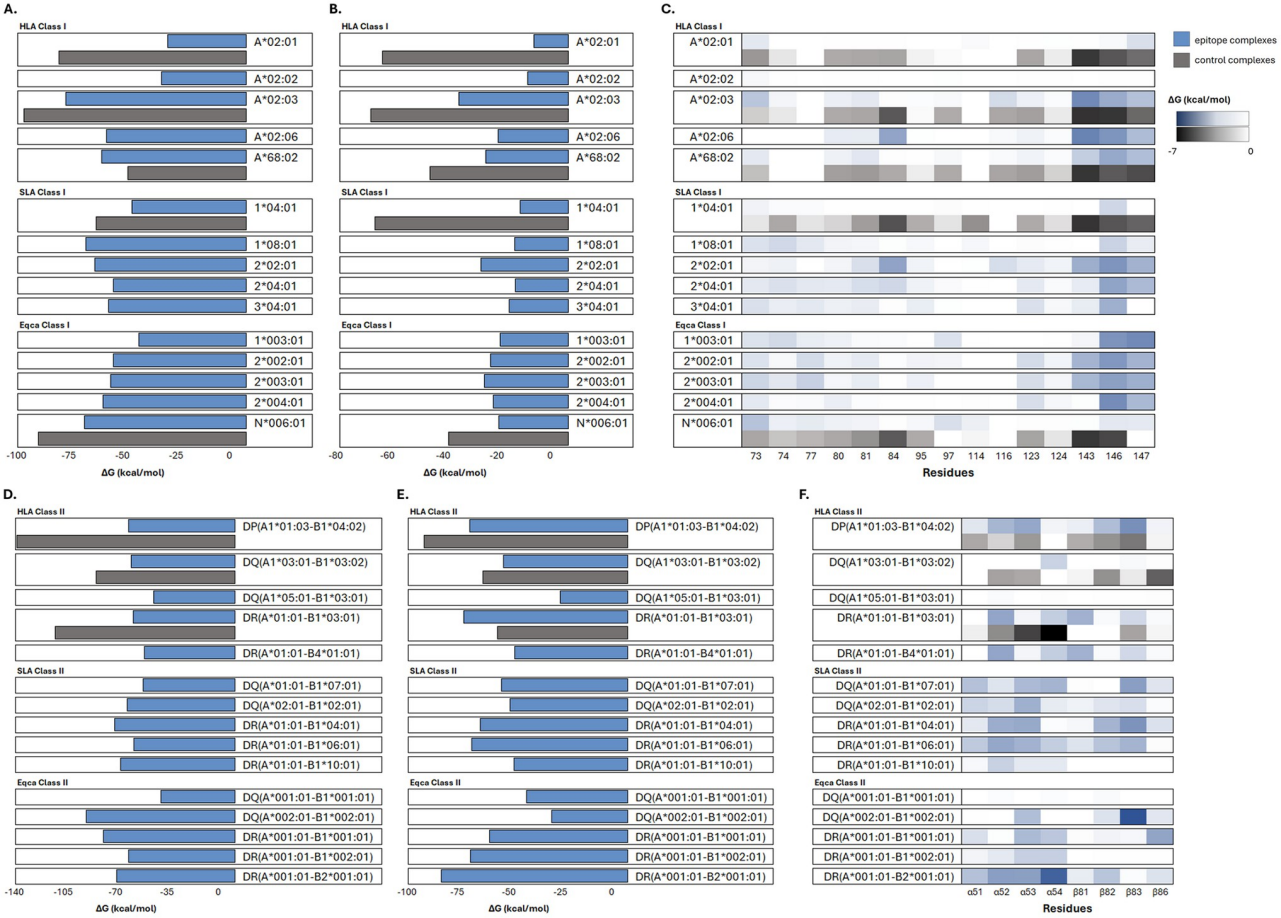
Relative binding free energies and energy decomposition analyses for CTL epitopes (A-C) and HTL epitopes (D-F), along with their respective control complexes. Epitope complexes are colored blue, whereas control complexes are represented in gray. Two sets of free energy calculations were performed using MM/GBSA method: pre-molecular dynamics using Hawkdock (**A,D**) and post-molecular dynamics using the gmxMMGBSA tool (**B,E**). Energy decomposition within the MHC residues involved in peptide binding (**C,F**).

Fig 2 shows the relative binding free energy and energy decomposition results of the CTL and HTL epitopes and control complexes. The binding affinity evaluation results of Hawkdock revealed that most of the predicted epitopes exhibited lower affinity to MHC molecules than the controls. This observation was further substantiated by the binding energy results obtained from the gmxMMGBSA tool after molecular dynamics simulation. However, despite the low average binding affinities, the residues of some epitopes exhibited strong affinities to some of the receptor residues, particularly within the anticipated binding regions.

Pocket F residues in MHC I [88], and pocket 1 residues in MHC II [88], are the most significant residues in peptide binding. The energy decomposition results suggest that certain epitope residues exhibited an increased capability to form strong interactions with these significant residues in the receptors. This localized, strong per-residue affinity could play a crucial role in the overall stability and effectiveness of the peptide-receptor interaction.

### Vaccine designing

Overlapping epitopes were fused into a consensus sequence to eliminate redundant fragments, thereby condensing the overall length of the construct [18]. This ensures that unique regions are represented in the final sequence, streamlining the overall structure without compromising the performance of individual epitopes, as antigen processing was previously considered.

Peptide linkers such as KK, AAY, GPGPG, HEYGAEALERAG, and EAAAK connected different components of the vaccine construct. The KK linker was used to connect individual LBL epitopes. Pairs of basic residues such as KK are the main cleavage sites of cathepsin B. This enzymatic cleavage is hypothesized to generate a neo-processable site that aids the release of LBL epitopes from a construct for presentation [107]. The AAY linker was used to connect CTL epitopes. Cleavage of AAY residues enables selective binding of C-terminal adjacent epitopes to chaperones or the TAP transporter [108], increasing the number of epitopes available for MHC binding and presentation. The GPGPG linker was used to connect individual HTL epitopes. Regions rich in glycine and proline are known to be associated with *β*-turns and the presence of this linker at ∼15–20 residue intervals is expected to contribute to the creation of secondary structures, potentially facilitating efficient cleavage during antigen processing [109]. The HEYGAEALERAG linker was used to connect the three epitope groups. This linker contains five specific recognition sites defined for proteasomes, which enhances the likelihood of efficient cleavage [110].

Immune response is a complex interplay of various factors and is not solely dictated by the presence of pathogen-specific antigens. When formulating vaccines, additional substances known as adjuvants are added to enhance the overall effectiveness of the immune response [13]. Various substances can serve as adjuvant components, but most subunit vaccines administered safely to humans have used endogenous adjuvants. This study extensively reviewed the existing literature to identify endogenous adjuvants that have demonstrated the ability to stimulate immune responses in laboratory settings (See Table 6).

**Table 6:** Endogenous adjuvants used in the study.

Adjuvant	Source	UniProt Entry	Target TLR	Ref
Resuscitation-promoting factor E (RpfE)	*Mycobacterium*	O53177	TLR4	[111]
Flagellin	*Salmonella*	Q06971	TLR5	[112]
50S ribosomal protein L7/L12 (50SrpL7/L12)	*Mycobacterium*	P9WHE3	TLR4	[113]
Phenol soluble modulin *α*4 (ps*α*4)	*Staphylococcus*	A9JX08	TLR2	[114]
Chorela toxin B (CTB)	*Vibrio*	P01556	TLR4	[115]
Heat shock protein (Hsp) 70_359–625_ (tHsp70)	humans	P9WMJ9	TLR4	[116]
*β*-defensin 3_23–67_ (h*β*d3)	humans	P81534	TLR1,2	[117]

**Fig 3:**
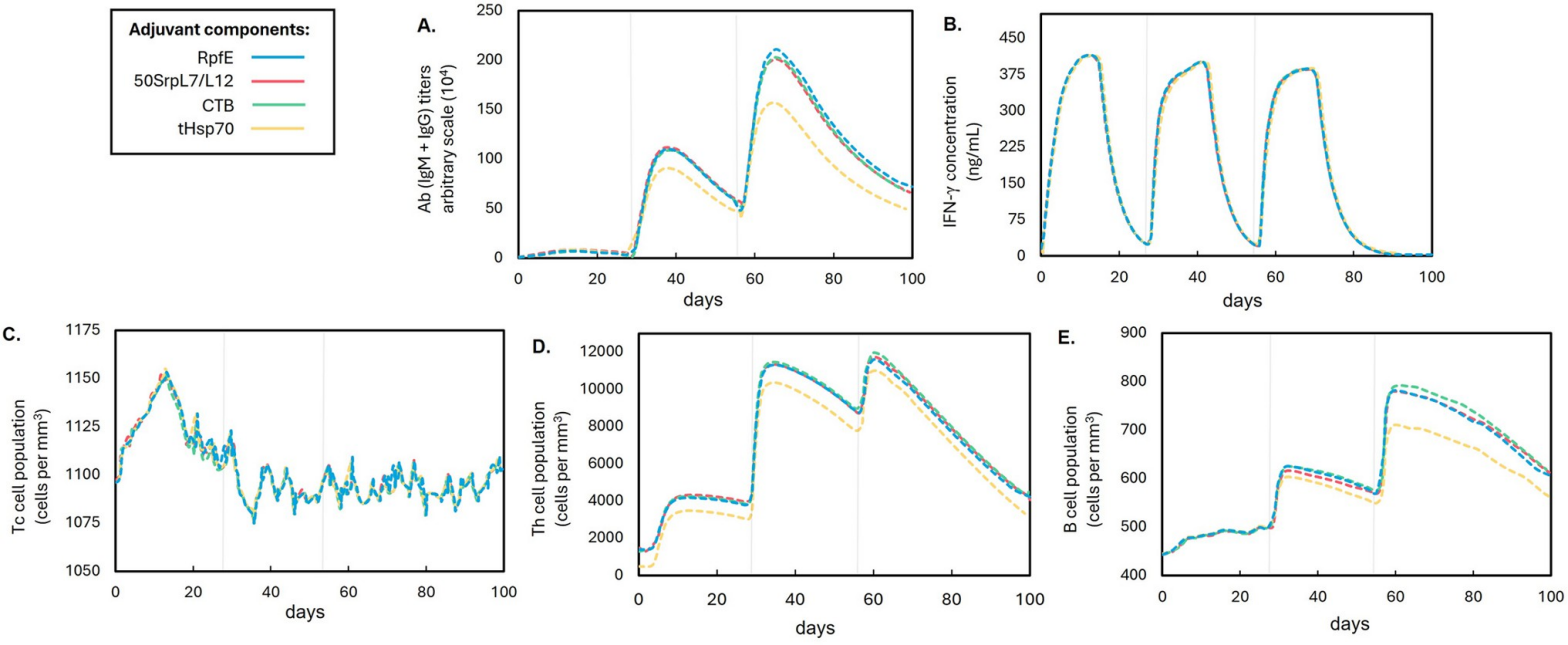
Comparison of the immune simulation profiles of the four vaccine constructs with different adjuvant formulations. Each formulation was differentiated using distinct colors. The gray line on the 28^th^ day indicates the second immunization while the gray line on the 56^th^ day indicates the third immunization. Graphs: (**A**) Antibody titers. (**B**) Interferon-*γ* concentration. (**C**) Cytotoxic T-lymphocyte (T_C_). (**D**) Helper T-lymphocyte (T_H_) populations. (**E**) B-lymphocyte populations.

**Fig 4:**
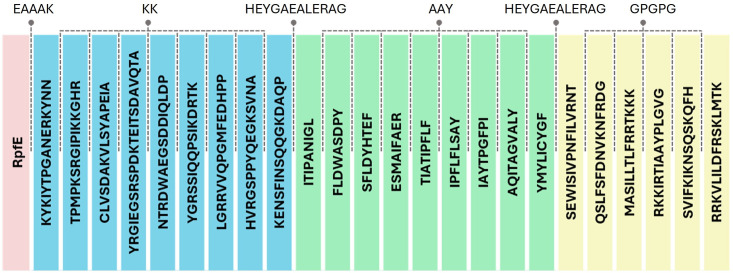
Graphical representation of the final vaccine construct components. This displays the identities and arrangement of the epitopes, adjuvants, and linkers.

The EAAAK linker connected the vaccine construct to the adjuvant sequences. This linker controls the distance and reduces the interference between the adjuvant and the vaccine construct [118], preventing steric hindrance or unwanted interactions that could compromise their structural integrity or functionality. Following an assessment of physicochemical properties (See Table 7), three of the seven vaccine candidates were insoluble upon overexpression to *E. coli*: constructs with flagellin, ps*α*4, and th*β*d3 adjuvants. These were eliminated from the list of vaccine candidates. Protein insolubility poses a challenge to vaccine production, as it typically results in the formation of inclusion bodies [73]; therefore, purification of these constructs is labor-intensive. C-ImmSim was used in the study to simulate the humoral and cellular responses of the mammalian immune system upon immunization of the four remaining vaccine constructs. The vaccine construct with the RpfE adjuvant was selected as the final vaccine construct due to its relatively higher immune response profile in C-ImmSim (See Fig 3). The configuration of the final vaccine construct with RpfE adjuvant is shown in Fig 4.

**Table 7:** Physicochemical properties of the vaccine constructs with different adjuvant formulations.

Vax-Adjuvant	Antigenicity Score^a^	Allergenicity	Instability Index^b^	Solubility^c^
Vax-RpfE	0.63	Non-allergen	39.18	Soluble
Vax-flagellin	0.56	Non-allergen	25.33	Insoluble
Vax-50SrpL7/L12	0.57	Non-allergen	31.93	Soluble
Vax-ps*α*4	0.60	Non-allergen	33.62	Insoluble
Vax-CTB	0.61	Non-allergen	35.22	Soluble
Vax-tHsp70	0.59	Non-allergen	32.82	Soluble
Vax-th*β*d3	0.63	Non-allergen	37.45	Insoluble

^a^ ≥0.4 designates a vaccine candidate antigenic;

^b^≤40 designates a vaccine candidate stable;

^c^upon overexpression in *E. coli*.

### Vaccine evaluation

#### Immune simulation and physicochemical tests

Six computationally designed multi-epitope subunit NiV vaccines retrieved from the literature [18, 20–22], were used as references to compare with the vaccine construct designed in this study. Recent vaccine designs were prioritized as they have incorporated the latest methods and benchmark studies in their analyses. This ensures that the designs used as references for comparison benefit from the advancements and improvements in immunoinformatics. S3 Table provides a detailed overview of the sequence, epitope configuration, and adjuvant used in the re NiV vaccine designs.

The physicochemical property evaluation was extended to the six reference vaccine designs (See Table 8). Along with the vaccine design of this study, the designs of Raju et al and the vaccine design 1 of Rahman et al passed all physicochemical property evaluations.

**Table 8:** Physicochemical properties of the NiV multi-epitope subunit vaccines.

Vax-Adjuvant	Antigenicity Score^a^	Allergenicity	Instability Index^b^	Solubility^c^
This study	0.63	Non-allergen	39.18	Soluble
Rahman et al. 1	0.59	Non-allergen	29.35	Soluble
Rahman et al. 2	0.67	Non-allergen	39.78	Insoluble
Srivastava et al. 1	0.44	Non-allergen	48.03	Insoluble
Srivastava et al. 2	0.48	Non-allergen	45.66	Soluble
Raju et al.	0.49	Non-allergen	27.07	Soluble
Majee et al.	0.64	Non-allergen	40.71	Insoluble

^a^ ≥0.4 designates a vaccine candidate antigenic;

^b^≤40 designates a vaccine candidate stable;

^c^upon overexpression in *E. coli*.

**Fig 5:**
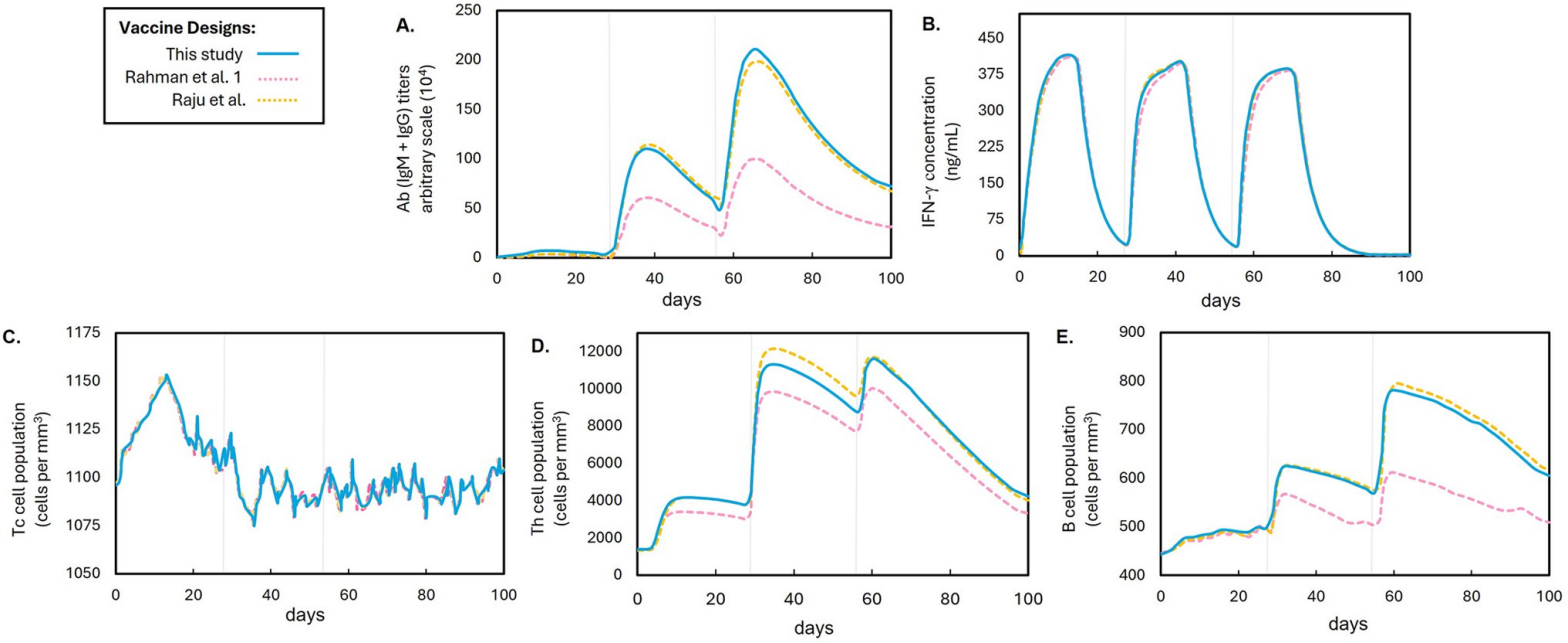
Comparison of the immune simulation profiles of the three multi-epitope subunit NiV vaccine designs. The straight blue line represents the NiV vaccine design of this study, while broken lines represent NiV vaccine designs from other studies, each differentiated by a distinct color. The gray line on the 28^th^ day indicates the second immunization while the gray line on the 56^th^ day indicates the third immunization. Graphs: (**A**) Antibody titers. (**B**) Interferon-*γ* concentration. (**C**) Cytotoxic T-lymphocyte (T_C_). (**D**) Helper T-lymphocyte (T_H_) populations. (**E**) B-lymphocyte populations.

C-ImmSim results showed no significant variations in interferon-*γ* concentrations and T_C_ lymphocyte populations across the three vaccine designs. However, vaccine design 1 of Rahman et al. exhibited a relatively low performance in inducing T_H_ lymphocyte populations, antibody titers, and B-lymphocyte populations (See Fig 5). Combining the findings from physicochemical property evaluations and immune simulation, the vaccine design of this study and that of Raju et al. emerged as the most viable designs, with the vaccine design of this study displaying a notably higher antigenicity score. Another key distinction in the vaccine design of this study lies in the consideration of other epitopes immunogenic to intermediate animal hosts of NiV, not limited to humans.

#### Tertiary structure assessment

**Fig 6:**
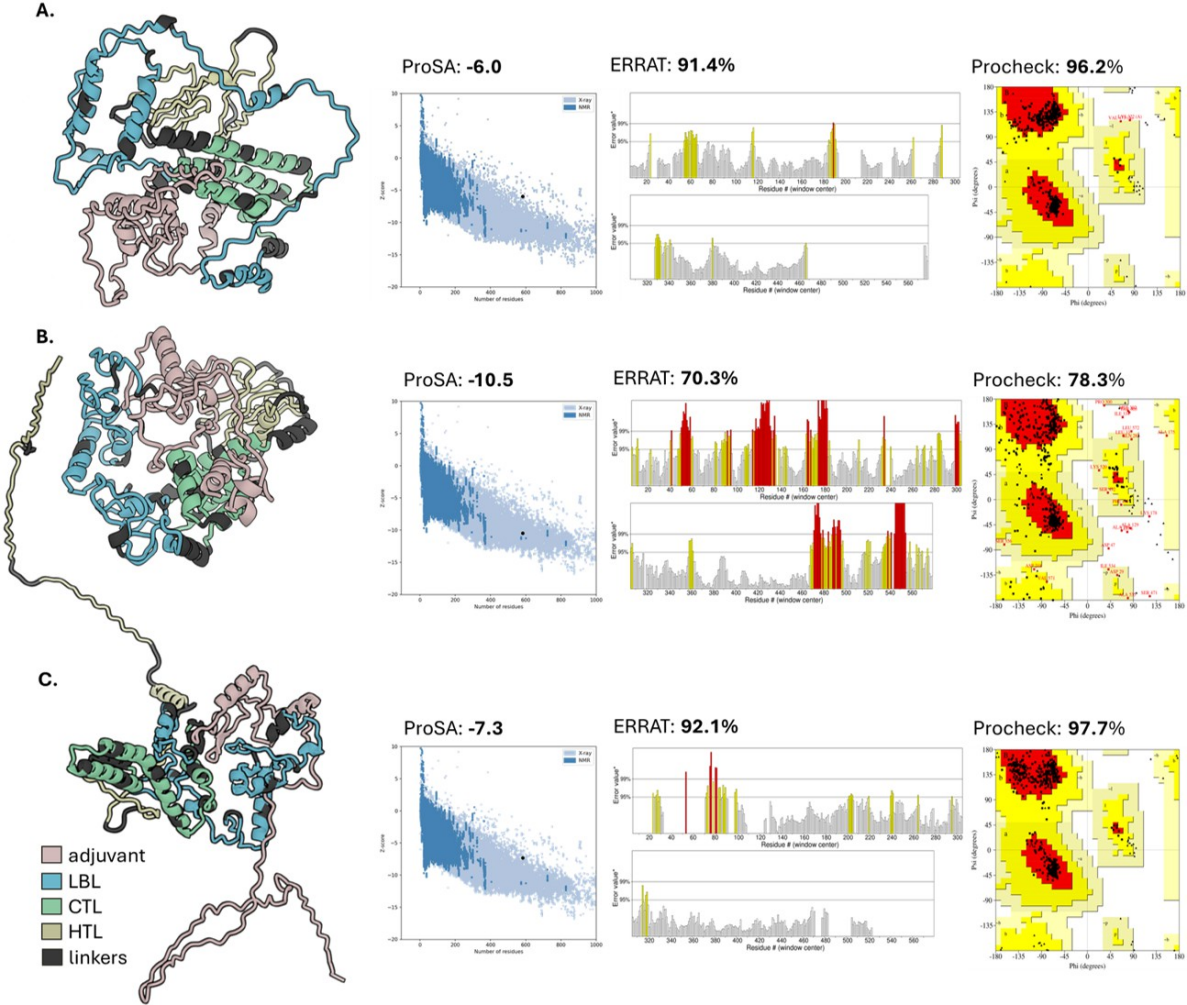
Comparison of the quality of the tertiary structure model predicted by (A) Alphafold, (B) D-ITASSER, and (C) Robetta. The adjuvant, epitope groups, and linkers are differentiated by distinct colors. In ProSA-web, a structure within the z-scores range indicates an accurate and reliable structure. A high-resolution structure in ERRAT produces 95% quality or higher and 91% for lower resolutions (2.5–3.0Å). A good quality structure in Procheck generally contains 90% residues in the most favorable regions.

Three modeling servers were used to predict the tertiary structure of the final vaccine construct. Utilizing multiple servers allows cross-validation, ensuring the reliability and accuracy of the predicted tertiary structure. Subsequent steps of minimization and refinement were performed to optimize and enhance the quality of the initially predicted models. Various quality assessment servers were used to assess the quality of the refined models. These servers evaluate different aspects of the structural models, such as stereochemistry, geometry, and overall structural compatibility. S4 Fig presents all structures of the refined models predicted by the three servers. The models that consistently exhibited the highest scores across the quality assessment servers are shown in Fig 6. The model generated by Robetta, which achieved the highest scores on ERRAT and Procheck, was used as the representative model for subsequent analysis.

#### Conformational B-lymphocyte epitope prediction

**Fig 7:**
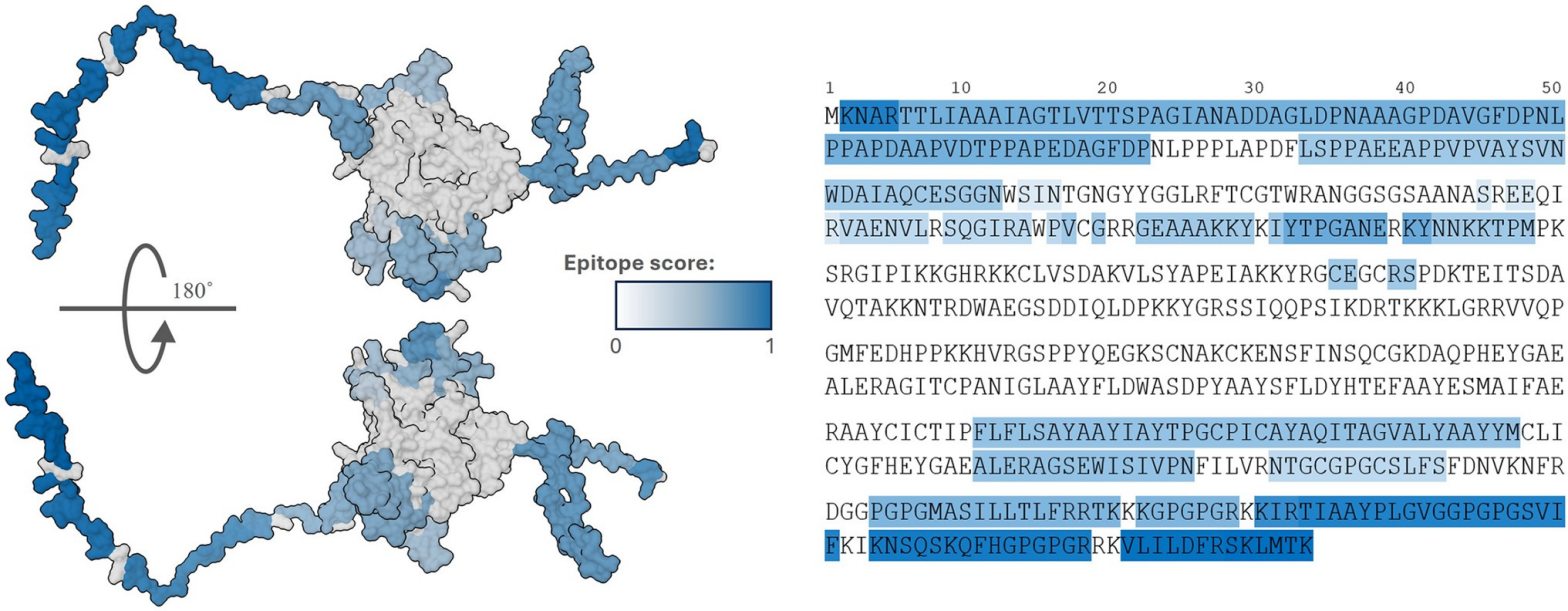
Conformational B-lymphocyte epitope regions within the vaccine construct predicted by the Ellipro tool. The darker the area, the higher the epitope score.

Fifteen conformational epitopes were predicted within the vaccine construct (See Fig 7). Notably, these epitopes were localized at the terminal ends, which are less constrained and more exposed than the tightly folded core. This increased exposure allows immune cells, such as B-lymphocytes, easier access to these regions, facilitating antibody binding and immune response activation [80]. Designing a vaccine construct rich in conformational epitopes is crucial, as approximately 90% of epitopes recognized by B-lymphocytes require specific conformations [119, 120].

#### Molecular docking and dynamics analyses of vaccine construct to TLR

TLR4 is a crucial component of the innate immune system [121–123]. It recognizes pathogen-associated molecular patterns and initiates immune responses. The activation of TLR4-related responses requires the binding of ligands, usually lipopolysaccharides (LPS) to TLR4 with myeloid differentiation factor 2 (MD2) [124]. This binding triggers conformational changes leading to homodimerization, and consequently, the production of proinflammatory cytokines [124]. Recently, the crystal structure of TLR4-MD2 bound to LPS was made available (PDB ID: 3FXI). This led to the derivation of computational simulation models that predict the interaction mechanism with the agonist. In addition to LPS, several endogenous agonists have been extensively studied and documented for their capacity to activate TLR4-related responses [125]. Given their ability to mimic the effects of LPS, albeit with varying potency and specificity degrees, these agonists have attracted attention as potential candidates for vaccine adjuvants.

**Fig 8:**
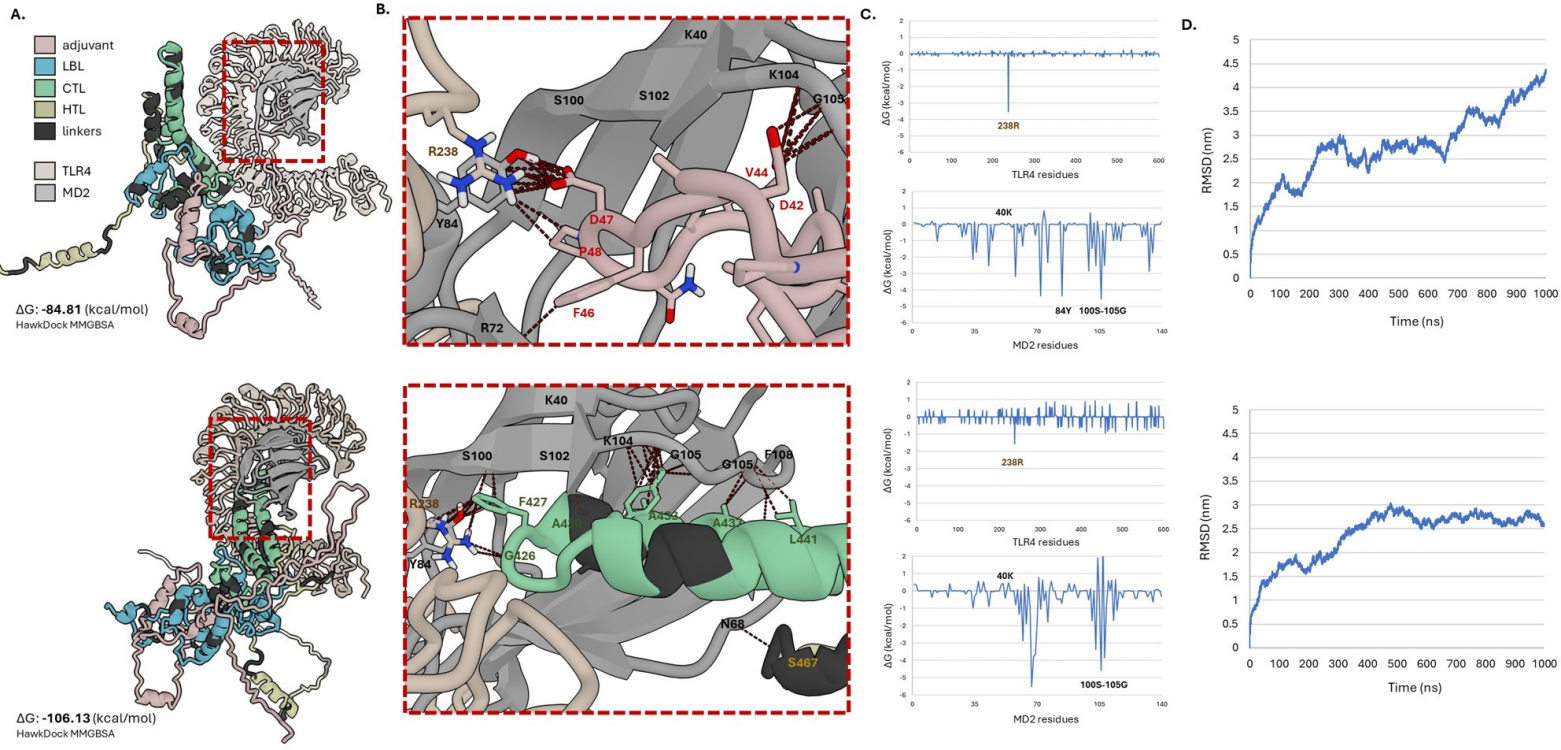
Molecular docking and dynamics analyses of the vaccine construct and TLR4-MD2. Two conformations of the binding, Complex 3 (top) and 7 (bottom) were shown. Figures: (**A**) Model of the complexes. (**B**) Model showing residues involved in the interaction of the complexes. (**C**) Per-residue binding free energies of TLR4 and MD2. (**D**) RMSD graph of the complexes following 1000 ns molecular dynamics simulations.

RpfE, the adjuvant used in the design, is a known TLR4 agonist [111, 125]. Molecular docking was conducted to visualize the possible interactions of the integrated RpfE in the vaccine construct with the TLR4-MD2 complex (See Fig 8). The representative model of the vaccine construct was docked to the structure of the TLR4-MD2 complex. The study used previously identified key residues of the TLR4-MD2 complex involved in the binding of LPS (R238 from TLR4; K40, Y84, S100, S102, K104, and G105 from MD2) to select the conformation of the complex that best mimics the agonist binding. From the ten possible conformations of the vaccine-TLR4-MD2 complexes predicted by the ClusPro server (See S5 Fig), only two complexes (C3 and C7) showed similar binding sites to LPS. For C3, a total of -84.81 kcal/mol free binding energy was recorded. Five of the binding sites (R238 from TLR4; Y84, S100, K104, and G105 from MD2), and additional residues (F58 and R72) were also observed to contribute to the overall binding free energy of the complex. Adjuvant residues (D42-P48) were observed to interact with the residues of the TLR4-MD2 complex. However, for C7, residues from the CTL epitopes (G426-L441) and HTL epitopes (S467-W469) formed the binding with the TLR4-MD2 complex. This complex has more negative free binding energy than C3, with -106.13 kcal/mol, and was one residue higher in terms of the interacted binding sites, with additional S102 from MD2. Additionally, the C7 complex maintained a stable binding to the TLR4-MD2 complex following a 1000 ns molecular dynamics simulation.

#### Disulfide engineering

**Fig 9:**
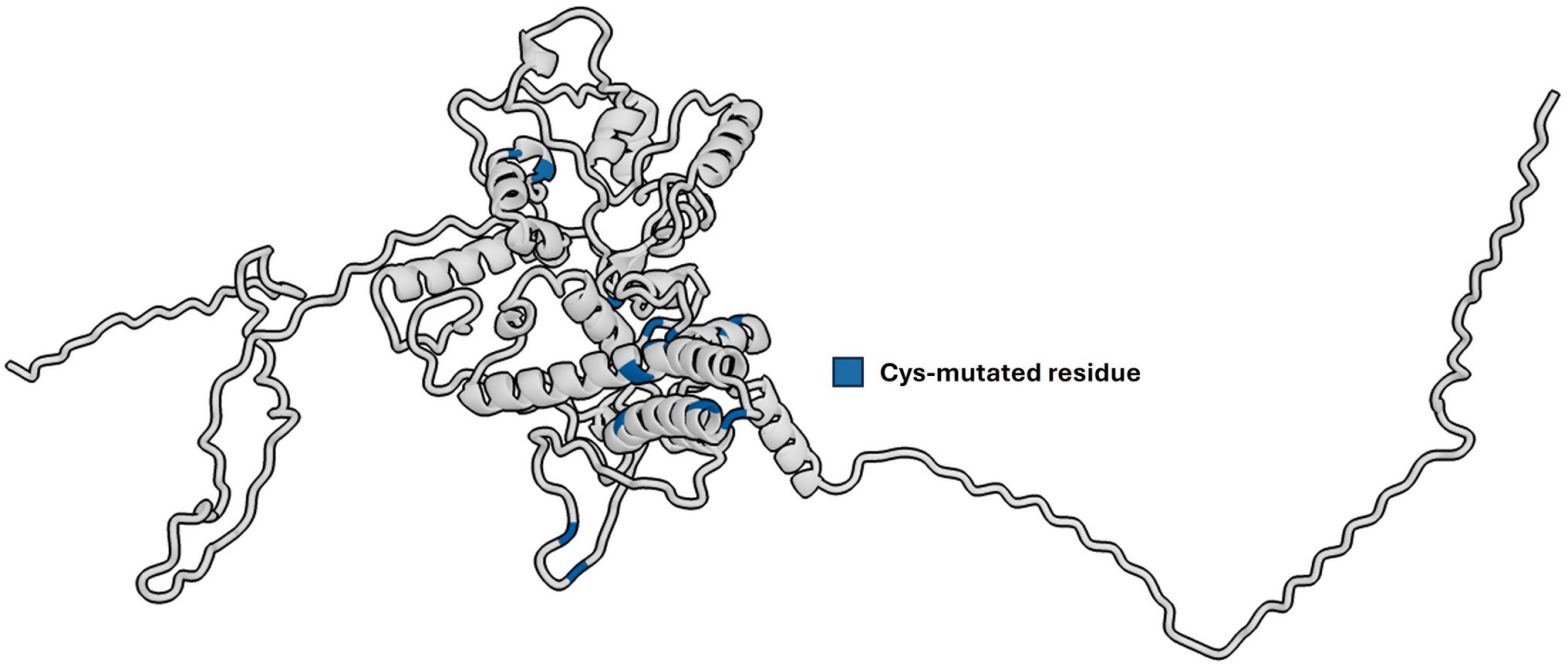
Cysteine-mutated residues within the vaccine construct. Residues suitable for mutation were identified using Disulfide by Design 2.0.

Disulfide by Design 2.0 identified seven residue pairs that can be mutated to cysteine, forming disulfide bonds (See Table 9). The specific locations of these residues are shown in Fig 9. As shown in S4 Table, the mutation of these residues led to a decrease in the instability index from 39.18 to 38.59, indicating an improvement in the overall stability of the construct. However, this modification also resulted in a slight decrease in antigenicity, from 0.63 to 0.61.

**Table 9:** Residues within the vaccine construct mutated to cysteine.

Residues	Identity
I235	LBL epitope
S238	LBL epitope
Q338	LBL epitope
A407	CTL epitope
A129	adjuvant
I359	CTL epitope
T405	CTL epitope
Y448	CTL epitope
F427	CTL epitope
A430	AAY linker
V324	LTL epitope
K328	KK linker
P484	GPGPG linker
Q488	HTL epitope

Further analysis, as presented in S6 Fig, compared the immune simulation results of the original construct to the construct with cysteine-mutated residues. The findings revealed no significant difference in IFN-*γ* concentration and T_C_ population. However, a relative increase in the T_H_ population, B-lymphocyte population, and antibody titers was recorded. Despite these favorable outcomes in immune simulation, it is important to note that the cysteine mutations may have affected the epitopes, potentially altering their specificity. It is recommended to use the construct with mutated residues if further laboratory experiments reveal that the original construct is unstable.

#### Codon optimization and *in silico* cloning

**Fig 10:**
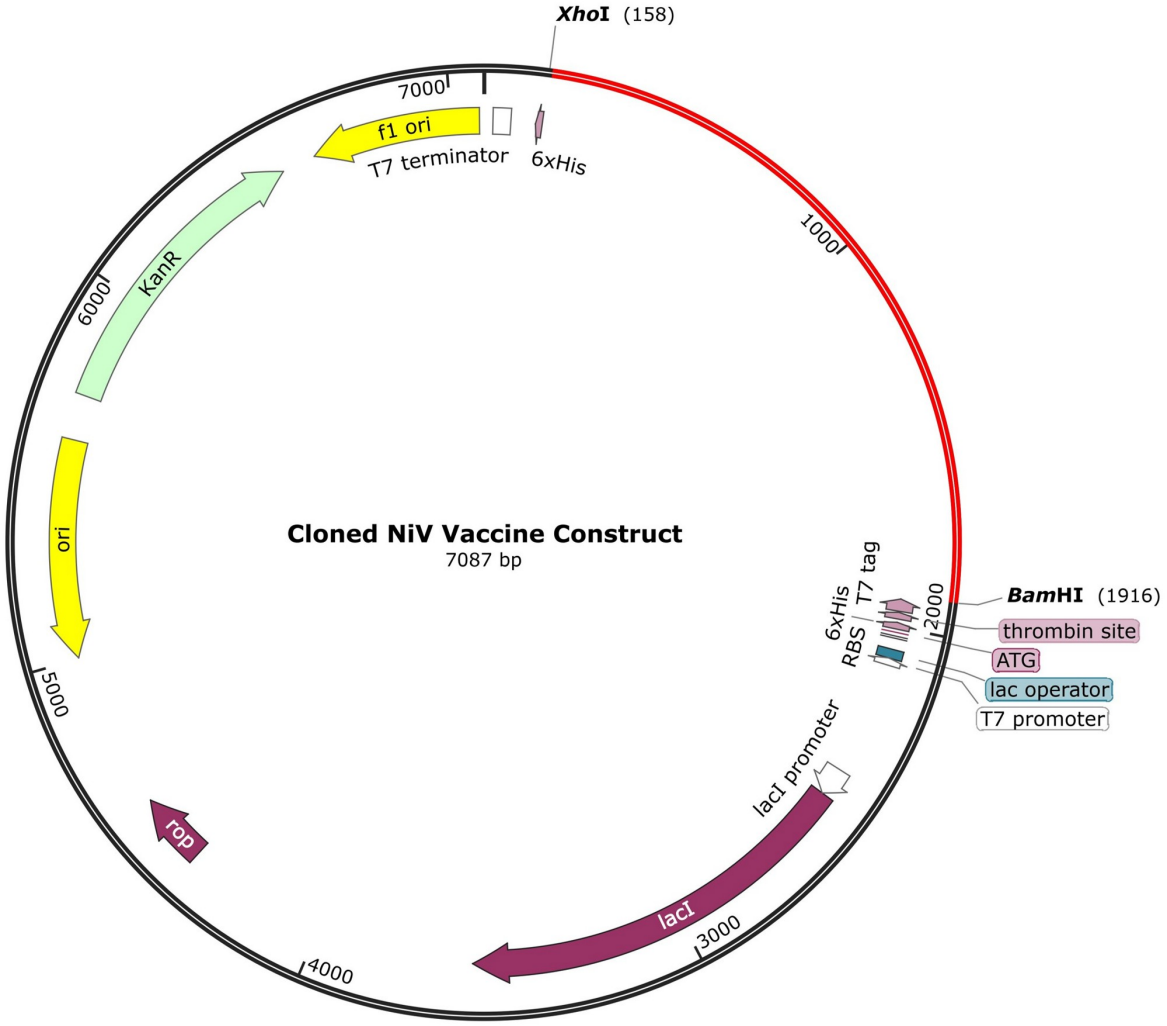
Cloned vaccine construct in a pET28(a)+ vector. The codon sequence is indicated in red while the plasmid backbone is shown in black.

The codon sequence generated by VectorBuilder had a GC content of 57.59% and a codon adaptation index (CAI) of 0.91. A sample of a cloned codon sequence of the vaccine construct in a pET28(a)+ vector is shown in Fig 10. The codon sequence was inserted into BamHI (164) and XhoI (158) restriction sites.

## Conclusion

This study used immunoinformatics and molecular modeling to design a multi-epitope subunit vaccine against NiV. This study is the pioneering multi-epitope vaccine design study to target not only humans but also intermediate animal hosts of NiV. Potential epitopes eliciting T and B-lymphocyte responses were mapped across all NiV proteins using the most updated and recently benchmarked computational servers. The screening process was implemented to ensure the immunogenicity and safety of the identified epitopes. Subsequently, molecular docking and dynamics analyses were conducted to validate the binding interactions of these epitopes with receptors in humans and the two animal hosts of the virus: swine and equine. Although low binding affinities to MHC molecules were observed among the predicted epitopes, certain regions within these epitopes demonstrated an increased capability to form strong interactions with specific receptor residues, potentially enhancing affinity in localized areas. Computational simulations revealed that the designed vaccine outperformed other multi-epitope vaccine designs against NiV in producing antibodies and activating T-lymphocytes. Although these computational methodologies demonstrate promising results, further experimental validation is needed to confirm the immunogenicity conferred by the designed vaccine and its epitope components. This study shows the capabilities of computational approaches in rational vaccine design and emphasizes the potential of a cross-species vaccination strategy as a proactive measure against the threat of NiV outbreaks.

## Supporting information

S1 Table Tertiary structure quality assessment scores of the major histocompatibility complex (MHC) Class I (A) and Class II (B) structures used for the docking and dynamics analyses. Good high resolution structures in ERRAT generally produce values around 95% or higher while 91% for lower resolutions (2.5-3.0Å). Good quality structures in Procheck generally have 90% residues in the most favorable regions.
(PDF)

S2 Table Control peptides used for docking and dynamics analyses of cytotoxic T-lymphocyte (A) and helper T-lymphocyte (B) epitopes.
(PDF)

S3 Table Recent multi-epitope subunit vaccine designs for Nipah virus available in the literature. (PDF)

S4 Table Physicochemical properties of the original and the cysteine-mutated vaccine constructs designed in this study. (PDF)

S1 Fig Sequence alignment of the major histocompatibility complex Class I (A) and Class II (B) molecules used in the study. The sequence above the alignment represents the predicted consensus sequence. The histogram above the consensus sequence indicates its conservancy. The alignment was generated using the program MAFFT and visualized through Jalview.
(PDF)

S2 Fig Dendrogram of the agglomerative hierarchical clustering of T-lymphocyte (A) and helper T-lymphocyte (B) epitope models with control peptides. Models of the control peptides are displayed in red. Epitope models clustering at RMSD (Å) height of 20 are colored in blue.
(PDF)

S3 Fig Graphical representation of the binding free energy of the docked major histocompatibility complex Class I-T-lymphocyte epitopes (A) and Class II-helper T-lymphocyte epitope (B) complexes. Scores of the control peptides are displayed in red while epitope models are displayed in gray.
(PDF)

S4 Fig Tertiary structure models of the multi-epitope subunit vaccine for Nipah virus designed in the study. Vaccine components are differentiated by distinct colors. The lettering in the names of the models is derived from the prediction servers (A: Alphafold, D: D-ITASSER, R: Robetta).
(PDF)

S5 Fig Models of the multi-epitope subunit vaccine for Nipah virus docked to the TLR4-MD2 complex. Vaccine components are differentiated by distinct colors. Blocks indicated the significant residues from the LPS-TLR4-MD2 complex interacted with the vaccine construct.
(PDF)

S6 Fig Comparison of the immune simulation profiles of the original and the cysteine-mutated vaccine constructs designed in this study. The straight blue line represents the original design while broken lines represent the cysteine-mutated design. The gray line on the 28^th^ day indicates the second immunization while the gray line on the 56^th^ day indicates the third immunization. Graphs: (**A**) Antibody titers. (**B**) Interferon-*γ* concentration. (**C**) Cytotoxic T-lymphocyte (T_C_). (**D**) Helper T-lymphocyte (T_H_) populations. (**E**) B-lymphocyte populations.
(PDF)
