# Supplementary material for: Advancing one health vaccination: In silico design and evaluation of a multi-epitope subunit vaccine against Nipah virus for cross-species immunization using immunoinformatics and molecular modeling
Source: PLoS One. 2024 Sep 26;19(9):e0310703. doi: 10.1371/journal.pone.0310703 (PMC11426463; doi:10.1371/journal.pone.0310703)
Supplement: S1 Table — Tertiary structure quality assessment scores of the major histocompatibility complex (MHC) Class I (A) and Class II (B) structures used for the docking and dynamics analyses. Good high resolution structures in ERRAT generally produce values around 95% or higher while 91% for lower resolutions (2.5-3.0Å). Good quality structures in Procheck generally have 90% residues in the most favorable regions. (PDF) [file pone.0310703.s001.pdf]

S1 TABLE. Tertiary structure quality assessment scores of the major histocompatibility complex (MHC) Class I (A) and Class II (B) structures used for the docking and dynamics analyses. Good high resolution structures in ERRAT generally produce values around 95% or higher while 91% for lower resolutions (2.5-3.0 Å). Good quality structures in Procheck generally have 90% residues in most favorable regions.

A.

**MHC Class I**	**ERRAT Score**	**Procheck Score**
HLA-A*02:01	97.6	92.4
HLA-A*02:02	97.0	93.7
HLA-A*02:03	98.2	91.8
HLA-A*02:06	95.9	93.0
HLA-A*68:02	98.2	91.7
SLA-1*04:01	97.5	94.2
SLA-1*08:01	95.2	95.5
SLA-2*02:01	97.6	93.6
SLA-2*04:01	98.8	92.3
SLA-3*04:01	95.3	91.0
Eqca-1*003:01	99.1	96.0
Eqca-2*002:01	99.4	93.6
Eqca-2*003:01	98.8	94.2
Eqca-2*004:01	95.5	94.8
Eqca-N*006:01	98.8	91.0

B.

**MHC Class II**	**ERRAT Score**	**Procheck Score**
HLA-DP(A1*01:03-B1*04:02)	95.9	93.1
HLA-DQ(A1*03:01-B1*03:02)	93.8	93.8
HLA-DQ(A1*05:01-B1*03:01)	92.5	95.2
HLA-DR(A*01:01-B1*03:01)	95.8	95.9
HLA-DR(A*01:01-B4*01:01)	95.2	95.9
SLA-DQ(A*01:01-B1*07:01)	98.6	93.1
SLA-DQ(A*02:01-B1*02:01)	92.5	93.7
SLA-DR(A*01:01-B1*04:01)	93.7	93.2
SLA-DR(A*01:01-B1*06:01)	96.4	93.0
SLA-DR(A*01:01-B1*10:01)	96.4	96.5
Eqca-DQ(A1*001:01-B1*001:01)	97.9	91.5
Eqca-DQ(A1*002:01-B1*002:01)	97.9	91.5
Eqca-DR(A*001:01-B1*001:01)	94.5	95.2
Eqca-DR(A*001:01-B1*002:01)	94.4	91.8
Eqca-DR(A*001:01-B2*001:01)	95.1	93.2
