# Supplementary material for: Advancing one health vaccination: In silico design and evaluation of a multi-epitope subunit vaccine against Nipah virus for cross-species immunization using immunoinformatics and molecular modeling
Source: PLoS One. 2024 Sep 26;19(9):e0310703. doi: 10.1371/journal.pone.0310703 (PMC11426463; doi:10.1371/journal.pone.0310703)
Supplement: S2 Table — Control peptides used for docking and dynamics analyses of cytotoxic T-lymphocyte (A) and helper T-lymphocyte (B) epitopes. (PDF) [file pone.0310703.s002.pdf]

S2 TABLE. Control peptides used for docking and dynamics analyses of cytotoxic T-lymphocyte (A) and helper T-lymphocyte (B) epitopes.

A.

Control peptides	MHC Class I	Source organisms	PDB ID
LLFGYPVYV	HLA-A*02:01	Human T-cell leukemia virus type I	1AO7
FLPSDFFPSV	HLA-A*02:03	Hepatitis B virus	3OX8
TLTSCNTSV	HLA-A*68:02	Human immunodeficiency virus	4I48
NSDTVGWSW	SLA-1*04:01	Influenza A virus	3QQ3
CTSEEMNAF	Eqca-N*006:01	Equine infectious anemia virus	4ZUU

B.

Control peptides	MHC Class II	Source organisms	PDB ID
QAFWIDLFETIGGGSLV	HLA-DP(A1*01:03-B1*04:02)	*Homo sapiens*	4P57
SGEGSFQPSQENP	HLA-DQ(A1*03:01-B1*03:02)	*Triticum aestivum*	2NNA
GGIGSDNKVTRRGG	HLA-DR(A*01:01-B1*03:01)	*Aspergillus nidulans*	7N19
