# Supplementary material for: Advancing one health vaccination: In silico design and evaluation of a multi-epitope subunit vaccine against Nipah virus for cross-species immunization using immunoinformatics and molecular modeling
Source: PLoS One. 2024 Sep 26;19(9):e0310703. doi: 10.1371/journal.pone.0310703 (PMC11426463; doi:10.1371/journal.pone.0310703)
Supplement: S3 Table — (PDF) [file pone.0310703.s003.pdf]

S3 TABLE. Recent multi-epitope subunit vaccine designs for Nipah virus available in the literature.

Author/s (Year)	Adjuvant used	Epitope configuration	Sequence
Majee (2020)	human β-defensin	CTL-HTL-LBL	GIINTLQKYYCRVRGGRCAVLSCLPKEEQIGKCSTRGRK-<br>CCRRKKEAAAKSSYYIIVRVYFPILTEIQQAYIQELAAYAQPPYHWSIAA<br>YKTLIRTHIKDRELRSELIGYLNKAAYSLMLLYREIGPRAPYMVLLEESI<br>QTKFAPGGYPLLWSFAMGVATTIAAYQPSIPREFMIYDDVFIDNTGRIAA<br>YILSAFNTVIALLGSIVIIVEAAAKILMQYIKANSKFIGIPMGLPQSIALSSL<br>MVAQEAAAKIYVDLSSYYIIVRVYGGGGSNNGNVCLVSDAKMLSYAG<br>GGGSNEFQSDLNTIKSLMLLYGGGGSTEMRNLLSQSLSVRKFMGGGGS<br>HIKINGVISKRLFAQGGGGSVFYQASFSWDTMIKFGDGGGGSNNEFYYV<br>LCAVSTVGGGGGSMDEGYFAYSHLERIGSGGGGSMDEGYFAYSHLERI<br>GSCSGGGGSISQSGEQTLLMIDNTTCPTAKKTDVPGAGPKDSAVKEEPP<br>QKKKAKAAKEAASSNATDDPAISNKKVEDVERTPETGKRKKIRTIAKKT<br>NVWTPPNPNTVYHCSAVYNEAAAKCTGKSC
Raju (2021)	50S ribosomal L7/L12	CTL-HTL-LBL	MAKLSTDELLDAFKEMTLLELSDFVKKFEETFEVTAAAPVAVAAA-<br>GAAPAGAAVEAAEEQSEFDVILEAAGDKKIGVIKVVREIVSGLGLKEAK<br>DLVDGAPKPLLEKVAKEAADEAKAKLEAAGATVTVKEAAAKAMDEG<br>YFAYAAYAENPVFTVFAAYVGFLVRTEFAAYPEICWEGVYGPGPGQGD<br>TLYFPAVGFLVRTGPGPGPANIGLLGSKISQSTGPGPGDSKILSAFNTVIA<br>LLKKIALLGSIVIIVMNIKKRSTDNQAVKKSCSRGVSKKLVVNWRNNTV<br>ISRPGKKPLVVNWRNNTVISRP
Rahman (2022)	padre sequence	CTL-HTL-LBL	AKFVAAWTLKAAAEAAAKSLIDTSSTI-<br>AAYSLMMTRLAVAAYITIPANIGLAAYYFPAVGFLVAAYRLSIGSPSKA<br>AYMTRLAVKPKAAYQPVFYQASFAAYKPKLISYTLAAYRPKLFAVKIA<br>AYTEIGPKVSLAAYLLDTVNPSLAAYSLISMLSMIAAYSIVPNFILVAAYF<br>ILVRNTLIAAYKTVYVLTALAAYTELSLDLALAAYIEIGFCLITGPGPGD<br>AFLIDRINWISAGVGPGPGGVYNDAFLIDRINWIGPGPGVYNDAFLIDRI<br>NWISGPGPGDPVSNSMTIQAISQAGPGPGISIVPNFILVRNTLIGPGPGPNF<br>ILVRNTLISNIEGPGPGYYIIVRVYFPILTEIGPGPGIGFCLITKRSVICNQK<br>KSKPENCRLSMGIRPNSKKINWISAGVFLDSNQTAKKYRAQLASEDTNA<br>QKTIKKKQRIIGVGEVLDRGDEKKIGTEIGPKVSLIDTSSKKPVFYQASFS<br>WDTMIKFKKPLLAMDEGYFAYSHLEKKSSTITIPANIGLLGSKKKPLKIH<br>ECNISCPNPLPKKSNLVGLPNNICLQKTSKKQSGEQTLLMIDNTTCPKKY<br>IQELLPVSFNNDNSEKKSEWISIVPNFILVRNTKKYVLTALQDYINTNLVP<br>KKISCKQTELSLDLALSKKKEAAAKGIINTLQKYYCRVRGGRCAVLSCL<br>PKEEQIGKCSTRGRKCCRRKK
Rahman (2022)	padre sequence	random	AKFVAAWTLKAAAEAAAKIGTEIG-<br>PKVSLIDTSSTITIPANIGLLGSKPLKIHECNISCPNPLPSNLVGLPNNICLQ<br>KTSKPKLISYTLPLLAMDEGYFAYSHLEKQRIIGVGEVLDRGDESLMMT<br>RLAVKPKYFPAVGFLVSKPENCRLSMGIRPNSRLSIGSPSKQPVFYQASF<br>SWDTMIKFGVYNDAFLIDRINWISAGVINWISAGVFLDSNQTAYRAQLA<br>SEDTNAQKTIRPKLFAVKIGGGGSKTVYVLTALQDYINTNLVPISCKQTE<br>LSLDLALSKDPVSNSMTIQAISQAYYIIVRVYFPILTEIYIQELLPVSFNND<br>NSESEWISIVPNFILVRNTLIPNFILVRNTLISNIEIGFCLITIGFCLITKRSVI<br>CNQQSGEQTLLMIDNTTCPLLDTVNPSLISMLSMIEAAAKGIINTLQKYY<br>CRVRGGRCAVLSCLPKEEQIGKCSTRGRKCCRRKK
Srivastava (2023)	human β-defensin	CTL	GIGDPVTCLKSGAICHPVFCPRRYKQIGTCGLPGTKCCK-<br>KPEAAAKMMASILLTLFGGGGSAQITAGVALYGGGGSFALSNGVLFGG<br>GGSKYLSDLLFVFGGGGSMTIQAISQAFGGGGSAENPVFTVFGGGGSAV<br>YNNEFYYGGGGSLAMDEGYFAYGGGGSTVYHCSAVYGGGGSNYMYLI<br>CYGFGGGGSYMIPRTMLEFGGGGSEIISDIGNYGGGGSTPFVDSRAYGG<br>GGSYPALALNEFGGGGSLDPVVTDVVYGGGGSLVSDAKMLSYGGGGS<br>MPSDDFSNTFGGGGSVSDAKMLSYGGGGSAEFFSFFRTFGGGGSETDD<br>YNGIYGGGGSFPISRLFNMYGGGGSFPVMGNRIYGGGGSIATVYTWAY<br>GGGGSIMKKSFKAYGGGGSIPFLFLSAYGGGGSKWYECFLFWFGGGGS<br>KYYQIDQPFFGGGGSLETDDYNGIYGGGGSRLFNMYRSYGGGGSSQNL<br>LVTSYGGGGSSYFGLVLVCFGGGGSTSDLDFVIFYGGGGSYPECNNILFE<br>AAAKGIINTLQKYYCRVRGGRCAVLSCLPKEEQIGKCSTRGRKCCRRKK
Srivastava (2023)	human β-defensin	HTL	GIGDPVTCLKSGAICHPVFCPRRYKQIGTCGLPGTKCCKKPEAAAK-<br>VQMTYNWTQWLQTLYGGGGSDLALSKYLSDLLFVFGGGGSLALSKYL<br>SDLLFVFGGGGGSALSKYLSDLLFVFGPGGGGSLSKYLSDLLFVFGPNG<br>GGGSSKYLSDLLFVFGPNLGGGGSKYLSDLLFVFGPNLQGGGGSYLSDL<br>LFVFGPNLQDGGGGSASFSWDTMIKFGDVLGGGGSFSWDTMIKFGDVL<br>TVGGGGSGVYNDAFLIDRINWIGGGGSNDAFLIDRINWISAGGGGGSDA<br>FLIDRINWISAGVGGGGSAFLIDRINWISAGVFGGGGSFLIDRINWISAGV<br>FLGGGGSIPREFMIYDDVFIDNGGGGSFMIYDDVFIDNTGRIGGGGSLSS<br>DQVAELAAAVQEGGGGSSSDQVAELAAAVQETGGGGSSDQVAELAAA<br>VQETSGGGGSDQVAELAAAVQETSAGGGGSQVAELAAAVQETSAGGG<br>GGSNNGNVCLVSDAKMLSGGGGSNGNVCLVSDAKMLSYGGGGSGNV<br>CLVSDAKMLSYAGGGGSNVCLVSDAKMLSYAPGGGGSVCLVSDAKML<br>SYAPEGGGGSNIDNIHLLAEFFSFFGGGGSIDNIHLLAEFFSFFRGGGGSD<br>NIHLLAEFFSFFRTGGGGSNIHLLAEFFSFFRTFGGGGSIHLLAEFFSFFRT<br>FGGGGGSLELASFLMDRRVILPGGGGSELASFLMDRRVILPRGGGGSLA<br>SFLMDRRVILPRAGGGGSASFLMDRRVILPRAAGGGGSLDFVIFYASLT<br>YLRRGGGGSFVIFYASLTYLRRGIEAAAKGIINTLQKYYCRVRGGRCAV<br>LSCLPKEEQIGKCSTRGRKCCRRKKHHHHHH
