# Supplementary material for: Advancing one health vaccination: In silico design and evaluation of a multi-epitope subunit vaccine against Nipah virus for cross-species immunization using immunoinformatics and molecular modeling
Source: PLoS One. 2024 Sep 26;19(9):e0310703. doi: 10.1371/journal.pone.0310703 (PMC11426463; doi:10.1371/journal.pone.0310703)
Supplement: S4 Table — (PDF) [file pone.0310703.s004.pdf]

S4 TABLE. Physicochemical properties of the original and the cysteine-mutated vaccine constructs designed in this study.

Vaccine design	Instability index[a]	Antigenicity score[b]	Allergenicity	Solubility[c]
Original	39.18	0.63	Non-allergen	Soluble
Mutated	38.59	0.61	Non-allergen	Soluble

[a] ≤ 40 designates a vaccine candidate stable; [b] ≥ 0.4 designates a vaccine candidate antigenic; [c] upon overexpression in *E. coli*.
