# Supplementary material for: Advancing one health vaccination: In silico design and evaluation of a multi-epitope subunit vaccine against Nipah virus for cross-species immunization using immunoinformatics and molecular modeling
Source: PLoS One. 2024 Sep 26;19(9):e0310703. doi: 10.1371/journal.pone.0310703 (PMC11426463; doi:10.1371/journal.pone.0310703)

**S1 FIGURE. Sequence alignment of the major histocompatibility complex Class I (A) and Class II (B) molecules used in the study.** The sequence above the alignment represents the predicted consensus sequence. The histogram above the consensus sequence indicates its conservancy. The alignment was generated using the program MAFFT and visualized through Jalview.

**A.**

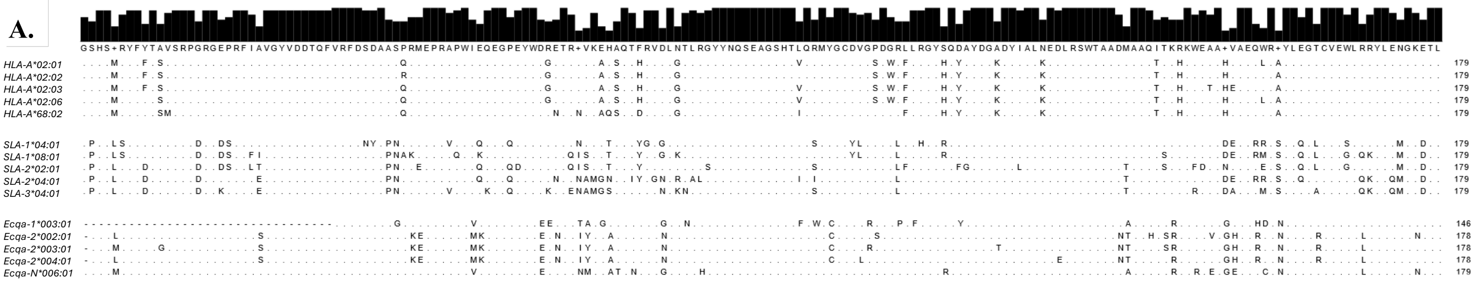


**B.**

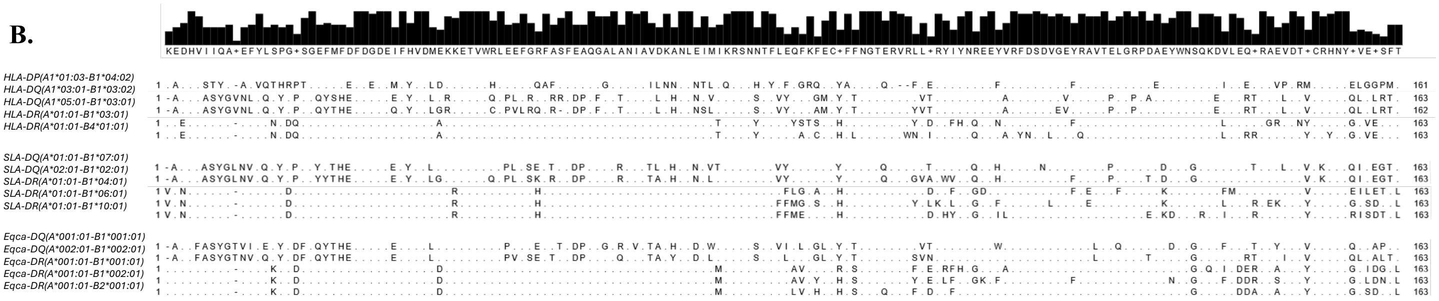

Supplement: S1 Fig — Sequence alignment of the major histocompatibility complex Class I (A) and Class II (B) molecules used in the study. The sequence above the alignment represents the predicted consensus sequence. The histogram above the consensus sequence indicates its conservancy. The alignment was generated using the program MAFFT and visualized through Jalview. (PDF) [file pone.0310703.s005.pdf]
