# Supplementary material for: Advancing one health vaccination: In silico design and evaluation of a multi-epitope subunit vaccine against Nipah virus for cross-species immunization using immunoinformatics and molecular modeling
Source: PLoS One. 2024 Sep 26;19(9):e0310703. doi: 10.1371/journal.pone.0310703 (PMC11426463; doi:10.1371/journal.pone.0310703)
Supplement: S2 Fig — Dendrogram of the agglomerative hierarchical clustering of T-lymphocyte (A) and helper T-lymphocyte (B) epitope models with control peptides. Models of the control peptides are displayed in red. Epitope models clustering at RMSD (Å) height of 20 are colored in blue. (PDF) [file pone.0310703.s006.pdf]

S2 FIGURE. Dendrogram of the agglomerative hierarchical clustering of T-lymphocyte (A) and helper T-lymphocyte (B) epitope models with control peptides. Models of the control peptides are displayed in red. Epitope models clustering at RMSD (Å) height of 20 are colored in blue.

A.

HLA-A*02:01

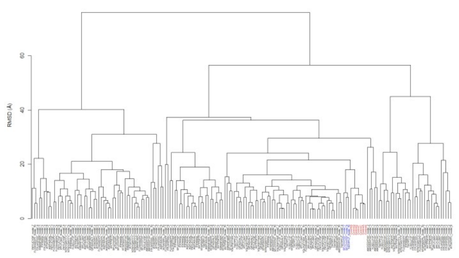

SLA-1*04:01

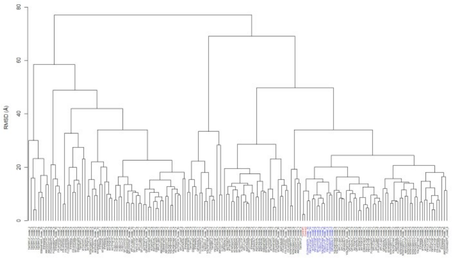

Eqca-1*003:01

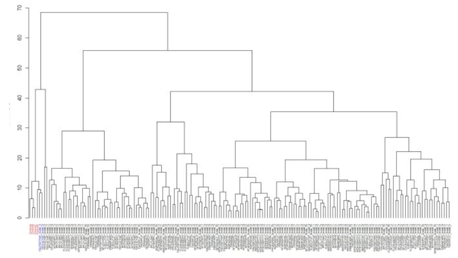

HLA-A*02:02

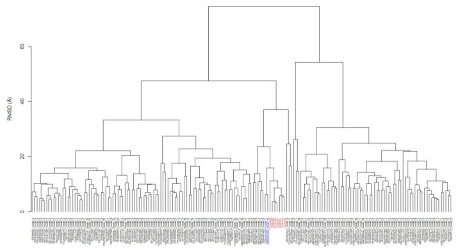

SLA-1*08:01

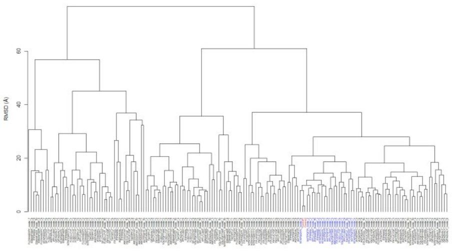

Eqca-2*002:01

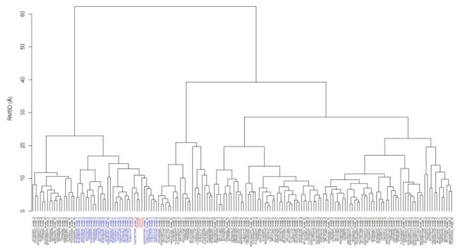

HLA-A*02:03

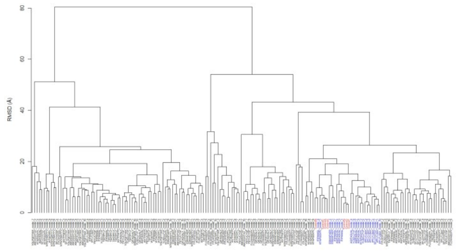

SLA-2*02:01

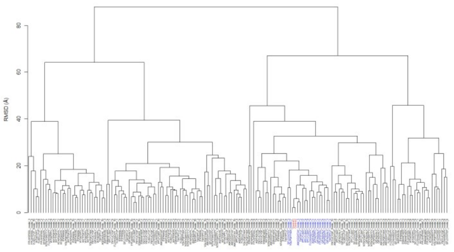

Eqca-2*003:01

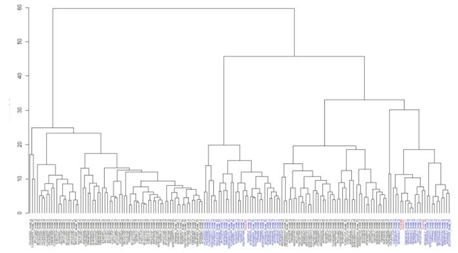

HLA-A*02:06

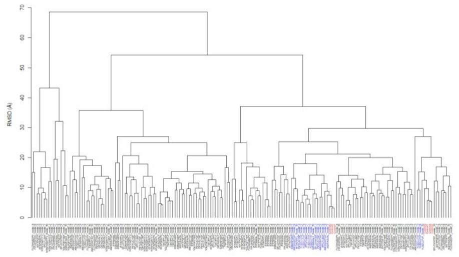

SLA-2*04:01

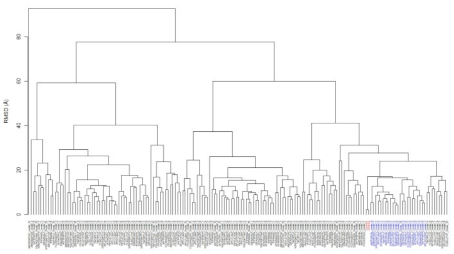

Eqca-2*004:01

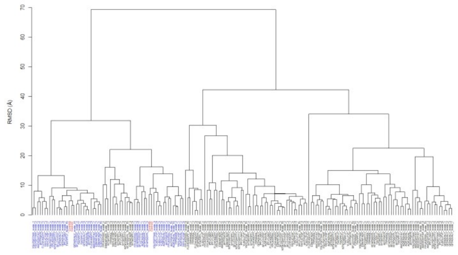

HLA-A*68:02

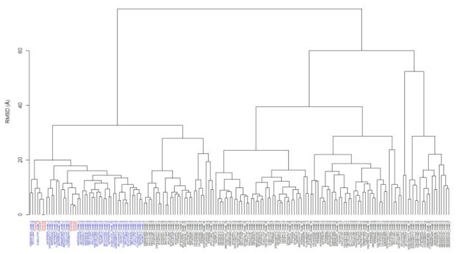

SLA-3*04:01

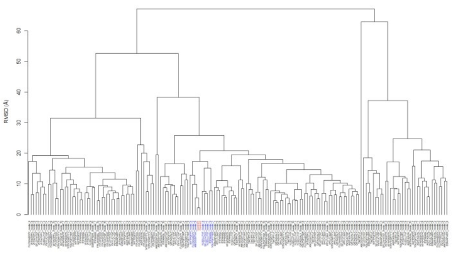

Eqca-N*006:01

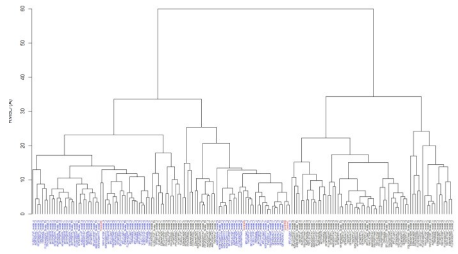

B.

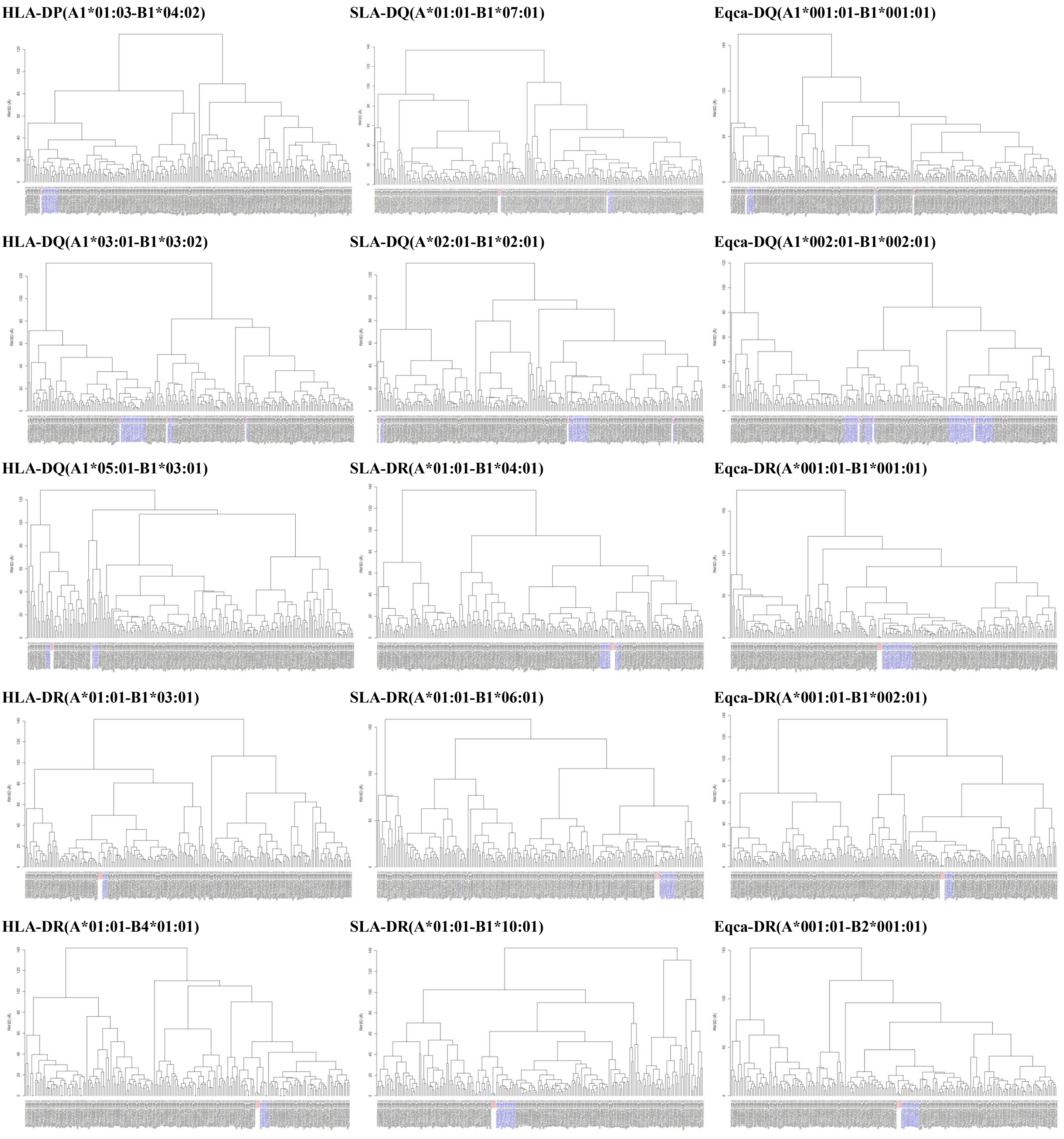
HLA-DP(A1*01:03-B1*04:02)
SLA-DQ(A*01:01-B1*07:01)
Eqca-DQ(A1*001:01-B1*001:01)
HLA-DQ(A1*03:01-B1*03:02)
SLA-DQ(A*02:01-B1*02:01)
Eqca-DQ(A1*002:01-B1*002:01)
HLA-DQ(A1*05:01-B1*03:01)
SLA-DR(A*01:01-B1*04:01)
Eqca-DR(A*001:01-B1*001:01)
HLA-DR(A*01:01-B1*03:01)
SLA-DR(A*01:01-B1*06:01)
Eqca-DR(A*001:01-B1*002:01)
HLA-DR(A*01:01-B4*01:01)
SLA-DR(A*01:01-B1*10:01)
Eqca-DR(A*001:01-B2*001:01)
