# Supplementary material for: Advancing one health vaccination: In silico design and evaluation of a multi-epitope subunit vaccine against Nipah virus for cross-species immunization using immunoinformatics and molecular modeling
Source: PLoS One. 2024 Sep 26;19(9):e0310703. doi: 10.1371/journal.pone.0310703 (PMC11426463; doi:10.1371/journal.pone.0310703)
Supplement: S3 Fig — Graphical representation of the binding free energy of the docked major histocompatibility complex Class I-T-lymphocyte epitopes (A) and Class II-helper T-lymphocyte epitope (B) complexes. Scores of the control peptides are displayed in red while epitope models are displayed in gray. (PDF) [file pone.0310703.s007.pdf]

S3 FIGURE. Graphical representation of the binding free energy of the docked major histocompatibility complex Class I-T-lymphocyte epitopes (A) and Class II-helper T-lymphocyte epitope (B) complexes. Scores of the control peptides are displayed in red while epitope models are displayed in gray.

A.

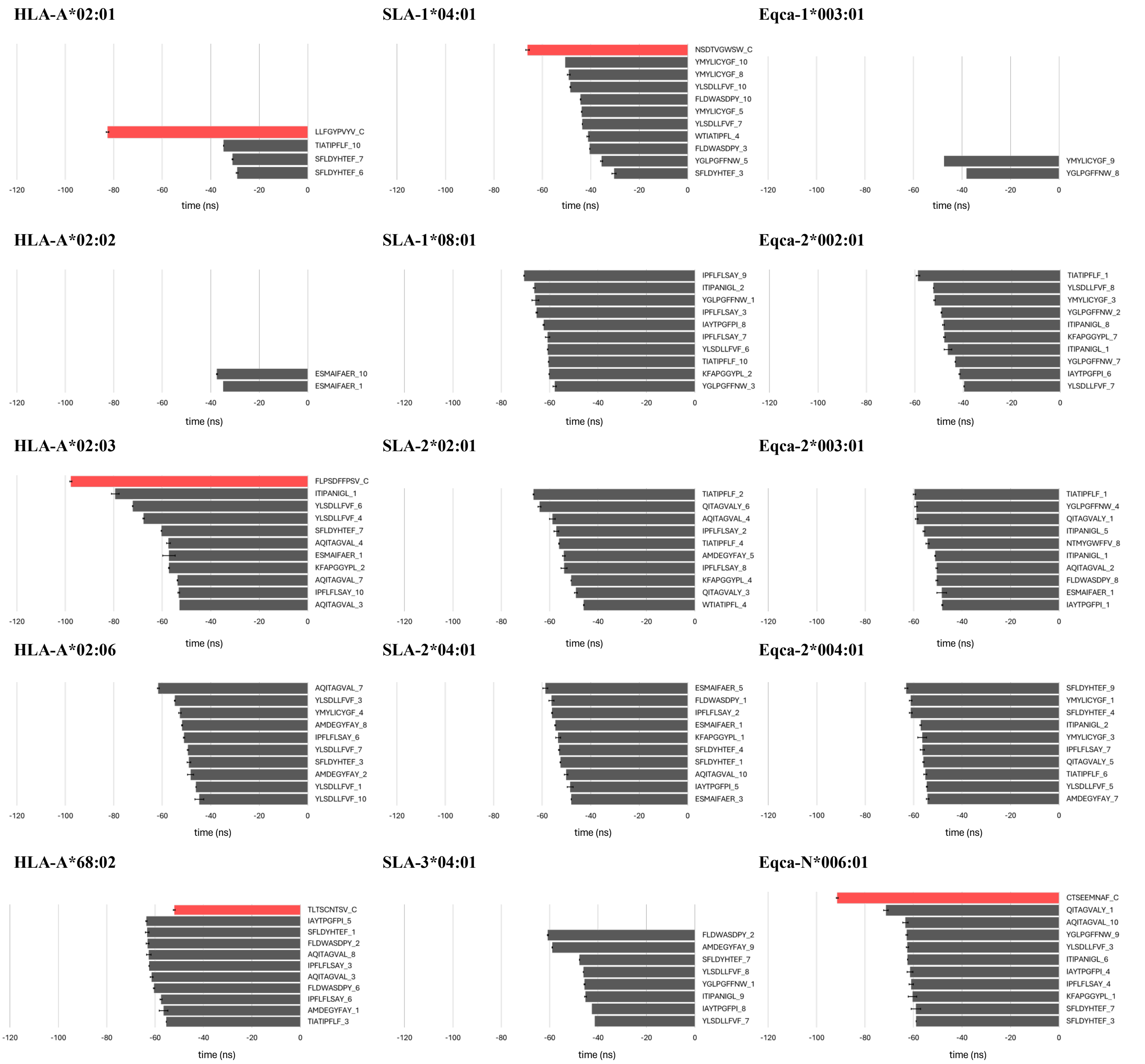

B.

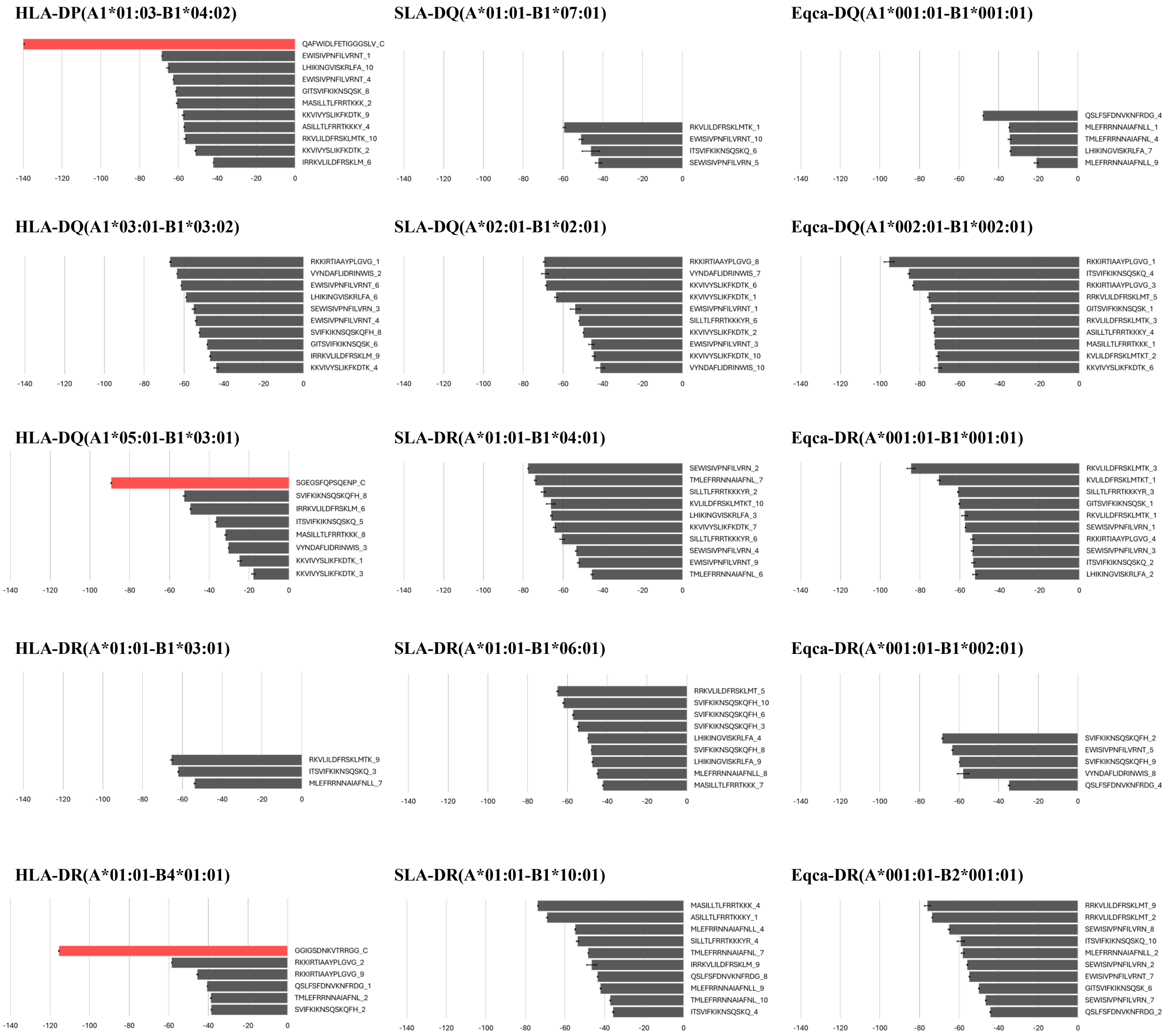
HLA-DP(A1*01:03-B1*04:02)
QAFWIDLFETIGGGSLV_C
EWISIVPNFILVRNT_1
LHIKINGVISKRLFA_10
EWISIVPNFILVRNT_4
GITSVIFKIKNSQSK_8
MASILLTLFRRTKKK_2
KKVIVYSLIKFKDTK_9
ASILLTLFRRTKKKY_4
RKVLILDFRSKLMTK_10
KKVIVYSLIKFKDTK_2
IRRKVLILDFRSKLM_6
SLA-DQ(A*01:01-B1*07:01)
RKVLILDFRSKLMTK_1
EWISIVPNFILVRNT_10
ITSVIFKIKNSQSKQ_6
SEWISIVPNFILVRN_5
Eqca-DQ(A1*001:01-B1*001:01)
QSLFSFDNVKNFRDG_4
MLEFRRNNAIAFNLL_1
TMLEFRRNNAIAFNL_4
LHIKINGVISKRLFA_7
MLEFRRNNAIAFNLL_9
HLA-DQ(A1*03:01-B1*03:02)
RKKIRTIAAYPLGVG_1
VYNDAFLIDRINWIS_2
EWISIVPNFILVRNT_6
LHIKINGVISKRLFA_6
SEWISIVPNFILVRN_3
EWISIVPNFILVRNT_4
SVIFKIKNSQSKQFH_8
GITSVIFKIKNSQSK_6
IRRKVLILDFRSKLM_9
KKVIVYSLIKFKDTK_4
SLA-DQ(A*02:01-B1*02:01)
RKKIRTIAAYPLGVG_8
VYNDAFLIDRINWIS_7
KKVIVYSLIKFKDTK_6
KKVIVYSLIKFKDTK_1
EWISIVPNFILVRNT_1
SILLTLFRRTKKKYR_6
KKVIVYSLIKFKDTK_2
EWISIVPNFILVRNT_3
KKVIVYSLIKFKDTK_10
VYNDAFLIDRINWIS_10
Eqca-DQ(A1*002:01-B1*002:01)
RKKIRTIAAYPLGVG_1
ITSVIFKIKNSQSKQ_4
RKKIRTIAAYPLGVG_3
RRKVLILDFRSKLMT_5
GITSVIFKIKNSQSK_1
RKVLILDFRSKLMTK_3
ASILLTLFRRTKKKY_4
MASILLTLFRRTKKK_1
KVLILDFRSKLMTKT_2
KKVIVYSLIKFKDTK_6
HLA-DQ(A1*05:01-B1*03:01)
SGEGSFQPSQENP_C
SVIFKIKNSQSKQFH_8
IRRKVLILDFRSKLM_6
ITSVIFKIKNSQSKQ_5
MASILLTLFRRTKKK_8
VYNDAFLIDRINWIS_3
KKVIVYSLIKFKDTK_1
KKVIVYSLIKFKDTK_3
SLA-DR(A*01:01-B1*04:01)
SEWISIVPNFILVRN_2
TMLEFRRNNAIAFNL_7
SILLTLFRRTKKKYR_2
KVLILDFRSKLMTKT_10
LHIKINGVISKRLFA_3
KKVIVYSLIKFKDTK_7
SILLTLFRRTKKKYR_6
SEWISIVPNFILVRN_4
EWISIVPNFILVRNT_9
TMLEFRRNNAIAFNL_6
Eqca-DR(A*001:01-B1*001:01)
RKVLILDFRSKLMTK_3
KVLILDFRSKLMTKT_1
SILLTLFRRTKKKYR_3
GITSVIFKIKNSQSK_1
RKVLILDFRSKLMTK_1
SEWISIVPNFILVRN_1
RKKIRTIAAYPLGVG_4
SEWISIVPNFILVRN_3
ITSVIFKIKNSQSKQ_2
LHIKINGVISKRLFA_2
HLA-DR(A*01:01-B1*03:01)
RKVLILDFRSKLMTK_9
ITSVIFKIKNSQSKQ_3
MLEFRRNNAIAFNLL_7
SLA-DR(A*01:01-B1*06:01)
RRKVLILDFRSKLMT_5
SVIFKIKNSQSKQFH_10
SVIFKIKNSQSKQFH_6
SVIFKIKNSQSKQFH_3
LHIKINGVISKRLFA_4
SVIFKIKNSQSKQFH_8
LHIKINGVISKRLFA_9
MLEFRRNNAIAFNLL_8
MASILLTLFRRTKKK_7
Eqca-DR(A*001:01-B1*002:01)
SVIFKIKNSQSKQFH_2
EWISIVPNFILVRNT_5
SVIFKIKNSQSKQFH_9
VYNDAFLIDRINWIS_8
QSLFSFDNVKNFRDG_4
HLA-DR(A*01:01-B4*01:01)
GGIGSDNKVTRRGG_C
RKKIRTIAAYPLGVG_2
RKKIRTIAAYPLGVG_9
QSLFSFDNVKNFRDG_1
TMLEFRRNNAIAFNL_2
SVIFKIKNSQSKQFH_2
SLA-DR(A*01:01-B1*10:01)
MASILLTLFRRTKKK_4
ASILLTLFRRTKKKY_1
MLEFRRNNAIAFNLL_4
SILLTLFRRTKKKYR_4
TMLEFRRNNAIAFNL_7
IRRKVLILDFRSKLM_9
QSLFSFDNVKNFRDG_8
MLEFRRNNAIAFNLL_9
TMLEFRRNNAIAFNL_10
ITSVIFKIKNSQSKQ_4
Eqca-DR(A*001:01-B2*001:01)
RRKVLILDFRSKLMT_9
RRKVLILDFRSKLMT_2
SEWISIVPNFILVRN_8
ITSVIFKIKNSQSKQ_10
MLEFRRNNAIAFNLL_2
SEWISIVPNFILVRN_2
EWISIVPNFILVRNT_7
GITSVIFKIKNSQSK_6
SEWISIVPNFILVRN_7
QSLFSFDNVKNFRDG_2
-140
-120
-100
-80
-60
-40
-20
0
