# Supplementary material for: Advancing one health vaccination: In silico design and evaluation of a multi-epitope subunit vaccine against Nipah virus for cross-species immunization using immunoinformatics and molecular modeling
Source: PLoS One. 2024 Sep 26;19(9):e0310703. doi: 10.1371/journal.pone.0310703 (PMC11426463; doi:10.1371/journal.pone.0310703)

S4 FIGURE. Tertiary structure models of the multi-epitope subunit vaccine for Nipah virus designed in this study. Vaccine components are differentiated by distinct colors. The lettering in the names of the models is derived from the prediction server (A: Alphafold, D: D-ITASSER, R: Robetta).

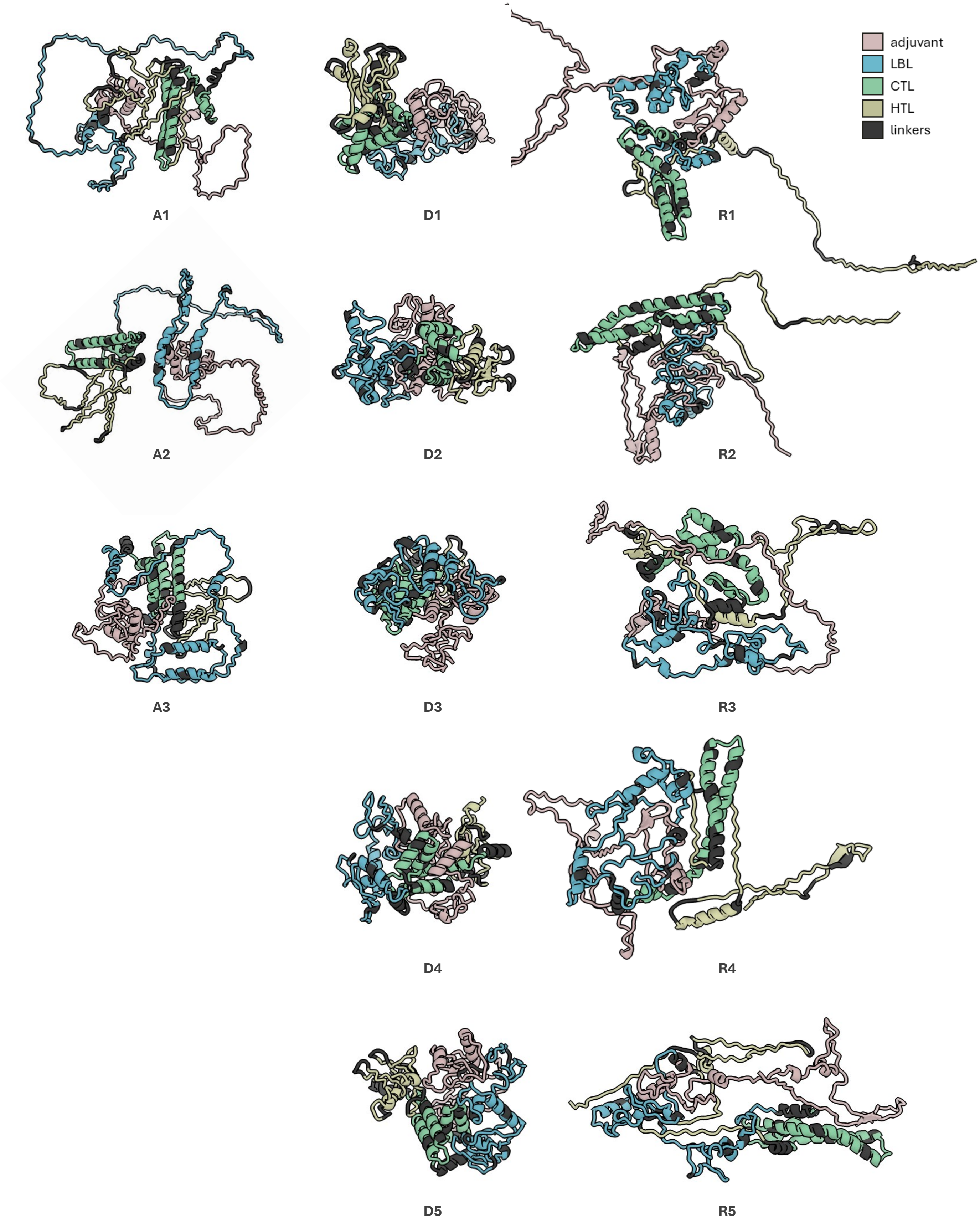

Supplement: S4 Fig — Vaccine components are differentiated by distinct colors. The lettering in the names of the models is derived from the prediction servers (A: Alphafold, D: D-ITASSER, R: Robetta). (PDF) [file pone.0310703.s008.pdf]
