# Supplementary material for: Advancing one health vaccination: In silico design and evaluation of a multi-epitope subunit vaccine against Nipah virus for cross-species immunization using immunoinformatics and molecular modeling
Source: PLoS One. 2024 Sep 26;19(9):e0310703. doi: 10.1371/journal.pone.0310703 (PMC11426463; doi:10.1371/journal.pone.0310703)

S5 FIGURE. Models of the multi-epitope subunit vaccine for Nipah virus docked to the TLR4-MD2 complex. Vaccine components are differentiated by distinct colors. Blocks indicated the significant residues from the LPS-TLR4-MD2 complex interacted with the vaccine construct.

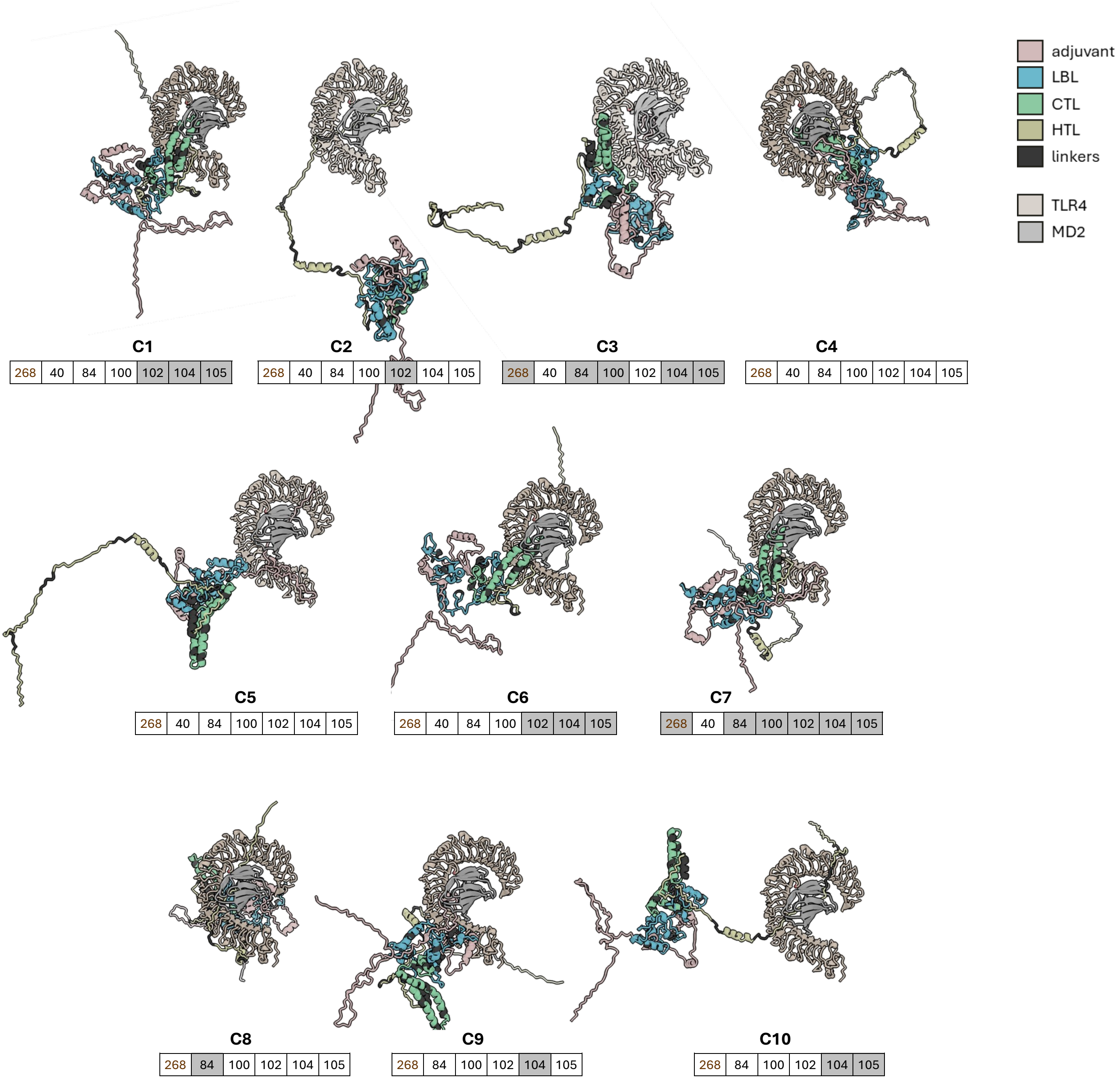

Supplement: S5 Fig — Vaccine components are differentiated by distinct colors. Blocks indicated the significant residues from the LPS-TLR4-MD2 complex interacted with the vaccine construct. (PDF) [file pone.0310703.s009.pdf]
