# Supplementary material for: Advancing one health vaccination: In silico design and evaluation of a multi-epitope subunit vaccine against Nipah virus for cross-species immunization using immunoinformatics and molecular modeling
Source: PLoS One. 2024 Sep 26;19(9):e0310703. doi: 10.1371/journal.pone.0310703 (PMC11426463; doi:10.1371/journal.pone.0310703)

**S6 FIGURE. Comparison of the immune simulation profiles of the original and the cysteine-mutated vaccine constructs designed in this study.** The straight blue line represents the original design while broken lines represent the cysteine-mutated design. The gray line on the 28th day indicates the second immunization while the gray line on the 56th day indicates the third immunization. Graphs: (**A**) Antibody titers. (**B**) Interferon-γ concentration. (**C**) Cytotoxic T-lymphocyte ($T_C$). (**D**) Helper T-lymphocyte ($T_H$) populations. (E) B-lymphocyte populations.

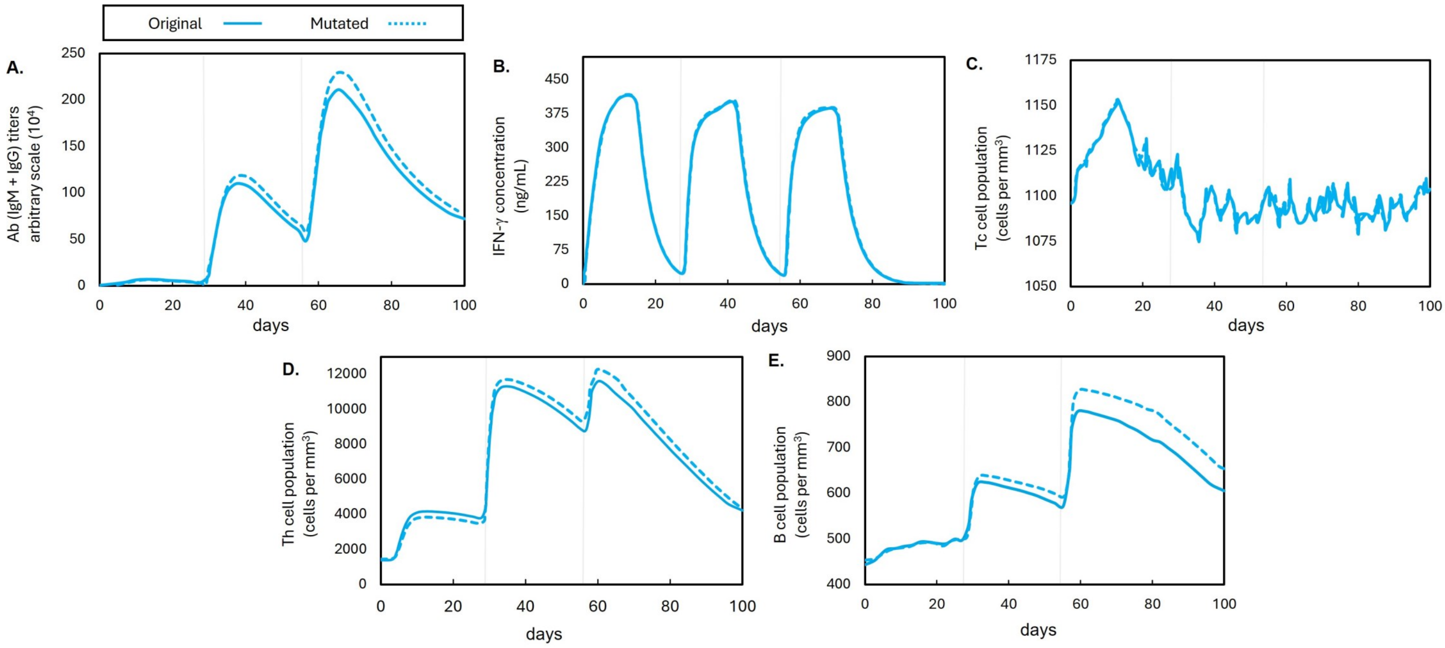

Supplement: S6 Fig — The straight blue line represents the original design while broken lines represent the cysteine-mutated design. The gray line on the 28th day indicates the second immunization while the gray line on the 56th day indicates the third immunization. Graphs: (A) Antibody titers. (B) Interferon-γ concentration. (C) Cytotoxic T-lymphocyte (TC). (D) Helper T-lymphocyte (TH) populations. (E) B-lymphocyte populations. (PDF) [file pone.0310703.s010.pdf]
